# Optimizing a tomato crocin biofactory by fine-tuning plant architecture

**DOI:** 10.3389/fpls.2025.1730399

**Published:** 2026-01-21

**Authors:** Maria Lobato-Gómez, Rafael Fernández-Muñoz, Diego Orzáez, Antonio Granell

**Affiliations:** 1 Instituto de Biología Molecular y Celular de Plantas, CSIC-Universidad Politécnica de València, Valencia, Spain; 2 Instituto de Hortofruticultura Subtropical y Mediterránea, IHSM CSIC-Universidad de Málaga, Algarrobo Costa, Málaga, Spain

**Keywords:** crocins, genome editing, plant architecture, saffron apocarotenoids, self-pruning, tomato fruit

## Abstract

Tomato (*Solanum lycopersicum*) has emerged as a promising platform for the sustainable production of high-value metabolites. In this study, we demonstrate that plant architecture remodeling via genome editing can be exploited as a chassis optimization strategy in plant biofactories. Building on the previously established Tomaffron line, which accumulates saffron apocarotenoids in the fruit, and based on the established knowledge that mutations in *SELF-PRUNING* (*SP*) and *SP5G* genes generate compact, determinate tomato plants, we used CRISPR/Cas9 to edit the *SP* and *SP5G* genes in Tomaffron to improve crocin production. The resulting *sp* sp*5g* double mutants exhibited a compact growth habit combined with significantly higher fruit yield, total crocin content, and firmer ripe fruits compared with non-mutants. Remarkably, crocin yields per square meter increased nearly fourfold compared to non-mutant Tomaffron plants grown at the same density, representing progress toward achieving the crocin yields of *Crocus sativus* and offering the advantage of easier cultivation and harvesting in the tomato system. Our results show that genome editing of plant architecture is not only a tool for agronomic improvement but also a powerful strategy to fine-tune our tomato biofactory performance, offering a scalable and sustainable approach for the production of valuable metabolites.

## 1 Introduction

Tomato (*Solanum lycopersicum*) is the second most widely cultivated horticultural crop, reaching a global production of 186.1 million tons in 2022 (FAOSTAT, 2024). It has been extensively used as a model organism due to its short life cycle, sympodial shoot development, fleshy fruit, the availability of a high-quality reference genome, and its vast phenotypic diversity, which is often well characterized genetically (Kimura and Sinha, 2008; Sato et al., 2012).

Tomato fruit is highly appreciated for its potent antioxidant activity, driven by the accumulation of high levels of carotenoids, ascorbic acid, flavonoids, and vitamin E (Frusciante et al., 2007). Furthermore, it serves as an excellent chassis for the metabolic engineering of exogenous pathways, benefiting from its several active endogenous biosynthetic pathways and the availability of various substrates. Transgenic tomato fruits with high levels of anthocyanins (Butelli et al., 2008), phenolic compounds (Luo et al., 2008), betalains (Polturak et al., 2017), and levodopa (Breitel et al., 2021) have been obtained expressing cDNAs encoding either transcription factors or biosynthetic enzymes driven by the E8 fruit ripening-specific promoter without causing pleiotropic effects on plant growth or fruit development (Li et al., 2018). The tomato cultivars most commonly used in these studies are Micro-Tom (Butelli et al., 2008; Luo et al., 2008; Polturak et al., 2017) and Moneymaker (MM) (Butelli et al., 2008; Luo et al., 2008; Breitel et al., 2021). Micro-Tom is a dwarf determinate tomato cultivar widely employed in research due to its short life cycle and small size (Meissner et al., 1997; Dan et al., 2006), while MM is an indeterminate commercial-type cultivar.

The production of high levels of saffron apocarotenoids has been achieved in tomato fruit through the expression of the *Crocus sativus* carotenoid cleavage dioxygenase (CCD2) under the control of the E8 promoter together with two glycosyltransferases under the control of a constitutive promoter. The best viable transgenic line from this work was named Tomaffron (Ahrazem et al., 2022). Saffron apocarotenoids, including crocins, picrocrocin, and safranal, are the most valuable metabolites in saffron spice. In addition to their coloring, flavoring and aroma properties exhibit high antioxidant activity and biological activity against many diseases. However, saffron is the most expensive spice due to the high manual labor required for its production (Cerdá-Bernad et al., 2022). One alternative to using *C. sativus* for obtaining these apocarotenoids would be their extraction from Tomaffron. Recently, it was demonstrated that crocins remain stable in Tomaffron-processed products, which retain the organoleptic properties of saffron even after one year of storage (Lobato-Gómez et al., 2025). The Tomaffron processing would be greatly complemented with a mechanical harvest of the fruits; however, the cultivar used for obtaining Tomaffron was MM, an indeterminate plant that does not allow mechanical harvesting, and this transgenic line contains the hygromycin resistance gene (*HygR*) as a selection marker for the transformation process (Ahrazem et al., 2022).

Maximizing metabolite yield per square meter is critical for scaling from laboratory production to commercial systems (Li et al., 2018). In indeterminate tomato cultivars, such as MM, the first inflorescence emerges after developing eight to twelve leaves, with subsequent inflorescences forming indefinitely every three leaves. This growth pattern is regulated by the opposing activities of flower-promoting (florigen) and flower-repressing (antiflorigen) signals. Research has demonstrated that mutations in the antiflorigen *SELF-PRUNING* (*SP*) gene accelerate inflorescence formation by removing florigen inhibition. This results in restricted shoot growth and a bushy, compact phenotype, which enhances uniform fruit ripening, a trait highly valued in the processing tomato industry (Pnueli et al., 1998; Gur et al., 2010; Park et al., 2014).

The *SP5G* gene has also exhibited antiflorigen activity, with tomato *sp5g* CRISPR/Cas9 mutants displaying accelerated flowering compared to wild-type plants while retaining indeterminate growth. Combining *sp* and *sp5g* mutations produces more compact plants than those with the single *sp* mutation, along with earlier growth determination and fruit yield (Soyk et al., 2017). These compact tomato plants, characterized by a burst of flowering and uniform fruit ripening, have significant agronomic value for the processing tomato industry (Eshed and Lippman, 2019) and are well-suited for mechanical harvesting in open-field plantations (Gur et al., 2010). The integration of these mutations in tomato biofactories could result in an earlier and higher production of valuable compounds accumulating in the tomato fruit.

In this work, we aimed to modify the plant architecture of Tomaffron to develop a more compact plant optimized for producing crocins, picrocrocin, and safranal, leveraging the knowledge of genes that enhance tomato suitability for the processing industry. To achieve these goals, the *SP*, *SP5G*, and *HygR* genes were independently mutated using CRISPR/Cas9 to assess the effect of each mutation on Tomaffron individually. Subsequently, the mutations were combined in a single plant. Our results demonstrate that while single *sp* or *sp5g* single mutants produced interesting phenotypes, the Tomaffron *sp* sp*5g* mutants exhibited a compact phenotype with earlier flowering and fruit ripening compared to non-mutated Tomaffron plants, along with a significant increase in fruit and crocin yield under two different environmental conditions, thus establishing a more efficient biotechnological production platform. Additionally, we generated tomato lines in which the hygromycin resistance gene used as a selectable marker in Tomaffron has been truncated to reduce the presence of non-plant antibiotic resistance DNA, thus addressing potential concerns regarding horizontal gene transfer.

## 2 Materials and methods

### 2.1 Plant material

Tomaffron, the best-performing transgenic line in terms of saffron apocarotenoid accumulation, yield, and viability from (Ahrazem et al., 2022) was used as the starting material for CRISPR/Cas9 editing experiments. The T0, T1, and T2 Tomaffron-edited plants were grown under greenhouse conditions at the IBMCP facilities in Valencia (2,808 hours of sunlight/year, 39.4698° N, 0.3774° W). The T3 edited plants were grown under greenhouse conditions at the IBMCP and IHSM La Mayora Experimental Station in Málaga (3,248 hours of sunlight/year, 36.7644° N, 4.0625° W).

At IBMCP, plants were transplanted to the greenhouse in November 2023 and fruit harvesting started in March 2024. The temperature was controlled by air conditioning, with a maximum of 24°C and a minimum of 21°C. In addition to natural light, the greenhouse had supplemental lighting with a photoperiod of 16 hours of light and 8 hours of darkness.

At La Mayora, plants were transplanted to the greenhouse in March 2024 and fruit harvesting started in mid-June 2024. The temperature was not controlled by air conditioning; instead, it was regulated by air convection through automated side windows and roof vents, and the temperatures were monitored. The average maximum/minimum temperatures recorded during the experiment were 30.1/12.9°C for the transplant and early growth period, 30.9/15.9°C for the fruit-setting period, and 36.5/19.7°C for the harvesting period. No supplemental lighting was used. At both locations, humidity was not controlled.

The T1-edited plants were cultivated for specific crossbreeding purposes. For this, some flowers were emasculated one day before anthesis and manually pollinated with pollen from donor plants collected on the same day the flowers opened.

### 2.2 Design and construction of the CRISPR/Cas9 vectors

The cDNA sequences of the *SELF-PRUNING* (SP, Solyc06g074350) and the *SELF-PRUNING 5G* (SP5G, Solyc05g053850) were retrieved from the Sol Genomics Network database (Fernandez-Pozo et al., 2015). The hygromycin resistance gene (*HygR*) cDNA sequence was obtained from the O1 construct in (Ahrazem et al., 2022).

**Figure 1:**
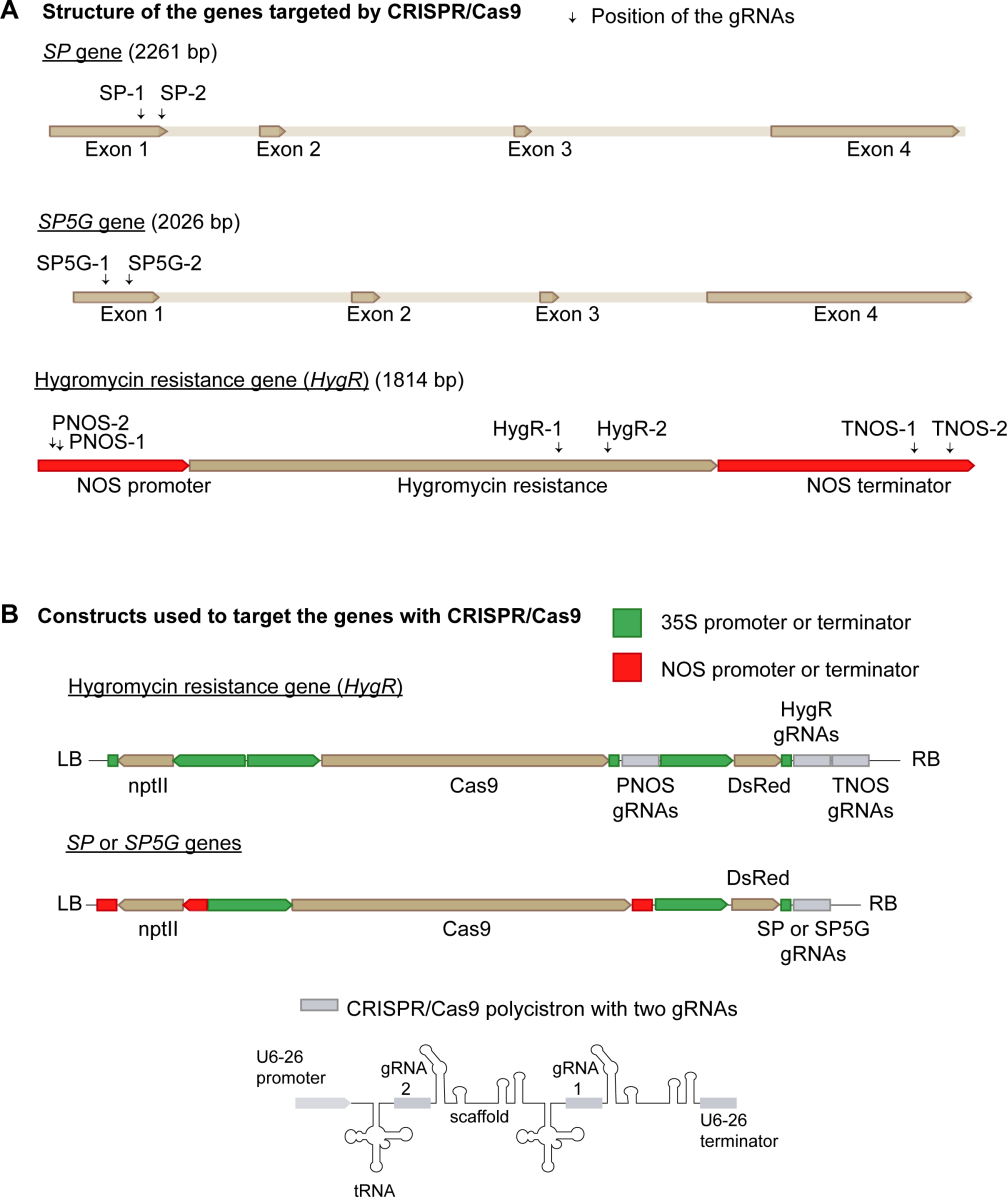
Targeted genes and constructs. **(A)** Representation of the exon regions of *SP* and *SP5G* genes, and the transcriptional unit of the hygromycin resistance gene (*HygR*). The positions of the designed guide RNAs (gRNAs) are indicated with arrows. **(B)** Schematic representation of the T-DNA of the constructs used to target *SP*, *SP5G*, and *HygR* with CRISPR/Cas9. *nptII*, kanamycin resistance gene; *SP*, self-pruning.

The guide RNAs (gRNAs) targeting the tomato genes were designed for *Streptococcus pyogenes* Cas9 using Benchling CRISPR Tool [Biology Software] (2022) with SL3.0 as the reference genome. The gRNAs with the highest on-target and lowest off-target scores were selected, and their potential off-targets were screened using the Cas-OFFinder algorithm using SL4.0 as reference genome (CRISPR RGEN Tools (rgenome.net)). None of the gRNAs selected had off-targets, according to the program. For each of the *SP* and *SP5G* genes, two gRNAs were designed. To induce large deletions in the *HygR* gene, a total of six gRNAs were designed: two gRNAs targeting the 5’ end region of the promoter (PNOS), two targeting the internal CDS, and two targeting the 3’ terminator (TNOS) regions flanking *HygR*. The gRNAs selected and used for this study are listed in **Supplementary Table 1**, and their locations in the genes are indicated in **Figure 1A**.

The construct design was performed as described in (Vázquez-Vilar et al., 2021) with slight variations. For the *HygR* targeting construct, new transcriptional units were designed to avoid using the NOS promoter and terminator (**Figure 1B**). All the cloning steps were carried out in *Escherichia coli*, and the final constructs were introduced into *Agrobacterium tumefaciens* LBA4404.

### 2.3 Tomaffron transformation and selection of edited lines

Tomaffron cotyledons were transformed as previously described (Ellul et al., 2003; Ahrazem et al., 2022). Briefly, tomato seeds were germinated *in vitro*, and ten-day-old cotyledons were inoculated with *A. tumefaciens* LBA4404 transformed with the construct of interest. After co-cultivation, the cotyledons were transferred to selection media. The shoots that developed roots were first screened for DsRed reporter signal to confirm transformation, and then leaf material was collected and used for DNA extraction.

Genomic DNA was extracted using CTAB and treated with RNase. The quality and concentration of the DNA were evaluated using a NanoDrop spectrophotometer (ThermoScientific, Massachusetts, USA). The mutations were screened by PCR followed by Sanger sequencing. The primers used to genotype the putative mutations are listed in **Supplementary Table 1**. The chromatograms were evaluated using the software Inference of CRISPR Edits (ICE) [Synthego Performance Analysis, ICE Analysis. 2019. v3.0. Synthego] (2023).

Edited lines from the T0 generation that exhibited the highest knock-out score and the highest percentage of 1- or 2-base pair indels were selected for further analysis in subsequent generations. To remove the edited construct, only T1 seeds lacking DsRed fluorescence were used to grow the T1 generation.

### 2.4 Plant and fruit phenotyping

Analyses of plant architecture, flowering, fruit ripening, and crocin accumulation were conducted using T3 plants of the single mutants (*sp* and *sp5g*) and F3 plants of the double mutants (*sp* sp*5g*) in greenhouses at IBMCP, Valencia (E1) and IHSM La Mayora, Málaga (E2). Untransformed Moneymaker (MM) was used as the control without crocins, while Tomaffron was the control with crocins.

At IBMCP, seeds were sown in small pots (15 cm diameter), and seedlings were transplanted into larger pots (25 cm diameter) after 18 days. The plants were grown under drip irrigation and mineral nutrition at 11.11 plants/m^2^ density. At La Mayora, seeds were sown in trays, and after 21 days, seedlings were transplanted into coconut-fiber grow slab bags with drip fertilization. The *sp* and *sp* sp*5g* mutants were grown either with two plants per sack (1.066 plants/m^2^) or one plant per sack (0.533 plants/m^2^), while the other genotypes were grown with three plants per sack (1.6 plants/m^2^). All plants were pruned in both experiments except Tomaffron *sp* and Tomaffron *sp* sp*5g* mutants. At La Mayora, Tomaffron *sp5g* single mutants were cultivated under pruned and unpruned conditions.

Flowering time was recorded by counting the number of leaves produced before the appearance of the first primary inflorescence and by noting the first anthesis flower on each plant. All anthesis flowers were labeled and recorded during one hundred days after the first anthesis. Ripening time was assessed by scoring the breaker stage in the fruits. After the emergence of the first breaker fruit, all breaker fruits emerging were tagged for 60 days. The number of inflorescences per plant was recorded.

At the IBMCP location, plant yield was assessed by weighing all red fruits per plant on day 115 of transplanting. Subsequently, fruits reaching the Br+10 stage were recorded daily for 15 days. At La Mayora, it was evaluated by separately weighing all red and green fruits per plant on day 129 after transplanting and counting the number of fruits at each ripening stage.

Fruit firmness was measured using the 53215 Fruit Hardness Tester (T.R. Turoni, Forlí, Italy), while objective color was assessed with the Portable Colorimeter CR-400 (Konica Minolta, Tokyo, Japan).

Crocins were extracted from 10 mg of freeze-dried tomato fruits following the protocol described in (Lobato-Gómez et al., 2024). The polar fraction was used to determine the crocin profile of Tomaffron by injection into the LC-MS as previously described (Ahrazem et al., 2022) and the total crocin accumulation by measuring absorbance at 443 nm, with the absorbance value of MM subtracted from that of Tomaffron fruits. Measurements were performed using an Infinite 200 Pro microplate reader (Tecan Group Ltd., Männedorf, Switzerland). The total crocin content was calculated using the crocin molar absorption coefficient (89000 L mol^-1^ cm^-1^) and the molecular weight of crocin-4 (976,972 g mol^-1^). Data from both environmental conditions were normalized to non-edited Tomaffron.

### 2.5 Gene expression analyses

RNA was extracted using the NucleoSpin RNA Kit (Macherey-Nagel, Düren, Germany). RNA was treated with DNase from the TURBO DNA-free™ Kit (Thermo Fisher, Massachusetts, USA), followed by cDNA synthesis using the PrimeScript™ 1st Strand cDNA Synthesis Kit (Takara, Shiga, Japan). Quantitative PCR (qPCR) was performed using TB Green™ Premix Ex Taq™ II (Takara) on a QuantStudio 3 (Thermo Fisher). Primers used are listed in **Supplementary Table 2**, with *S. lycopersicum* Clathrin Adaptor Complexes subunit (*CAC*) as the reference gene. *CAC* is a widely used housekeeping gene in *S. lycopersicum* (Wieczorek et al., 2013).

### 2.6 Statistical analysis

Statistical analyses were conducted using R (version 4.5.0) and graphs were generated using GraphPad Prism. The Mixomics package was used to perform Principal Component Analysis (PCA) (Florian et al., 2017), with scaling and centering of the data.

The choice of statistical tests was based on data distribution, variance heterogeneity, and sample size per genotype. When parametric assumptions were met, group differences were evaluated using one-way ANOVA followed by Tukey’s *post hoc* test. When variances were unequal, Welch’s one-way ANOVA with Games–Howell *post hoc* comparisons was applied.

For variables with small and/or unequal sample sizes, non-normal distributions, or pronounced outliers, group differences were assessed using a one-way permutation ANOVA with 10,000 resampling iterations. Significant effects were followed by pairwise permutation tests with false discovery rate correction (Benjamini–Hochberg).

Data are presented as mean ± SD and statistical significance was defined as *p* < 0.05 for analyses based on one-way ANOVA or Welch’s ANOVA. For permutation-based analyses, data are presented as median with interquartile range (25th–75th percentile), and statistical significance was defined as adjusted *p*-values (*p*-adj < 0.05). The statistical test applied for each analysis is specified in the corresponding figure legend.

## 3 Results

### 3.1 Selection of Tomaffron edited lines

**Figure 2:**
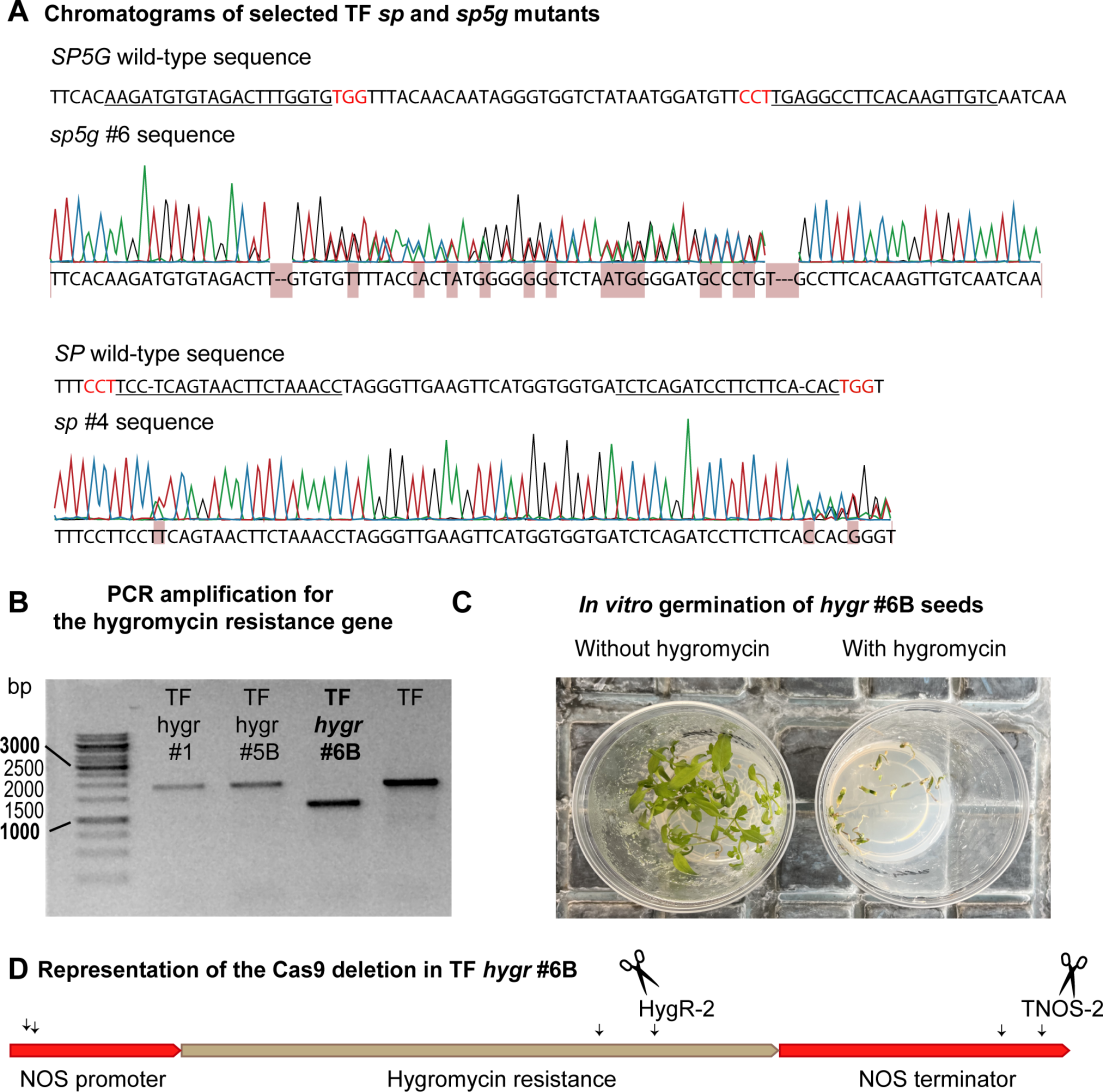
CRISPR/Cas9 editing results. **(A)** Chromatograms of the selected *sp* #*4* and *sp5g* #6 edited lines. The gRNA sequences are underlined, and the PAM sequences are highlighted in red. The mismatches between the wild-type and mutated sequences are indicated in red in the mutated sequence. **(B)** Agarose gel of the PCR for *HygR* gene amplification. **(C)** Results of *in vitro* germination of *hygr* #6B seeds with and without hygromycin in the media. **(D)** Representation of the large deletion in *hygr* #6B line. TF, Tomaffron; *SP*, self-pruning.

Tomaffron plantlets edited for the *SP*, *SP5G*, and *HygR* genes were identified among the DsRed-positive T0 plantlets by PCR and sequencing. Eight and three plants with high knock-out scores were recovered for *SP* and *SP5G*, respectively (**Supplementary Table 3**). One edited line for each targeted gene was selected for greenhouse experiments in the next generations. For *SP*, we selected the *sp* #4 line, which carries 1- and 2-base pair (bp) indels that would result in a frameshift of the coding sequence. For *SP5G*, the *sp5g* #6 mutant line, carrying a 5-bp indel, was selected (**Figure 2A**).

In the *HygR*-targeted lines, *hygr* #6B showed a large 617 bp deletion between HygR-2 and TNOS-2 gRNAs (**Figures 2B, D**). Hygromycin susceptibility was assessed by sowing seeds *in vitro* with or without hygromycin. Germination was 95% on media without hygromycin and 0% on media with it, confirming susceptibility (**Figure 2C**).

To move to the T2 generation, we screened the T1 seeds for the absence of DsRed to counterselect the seeds with mutations in the targeted genes that do not contain the CRISPR/Cas9 construct. Three random T1 plants from each mutant line were genotyped and exhibited a 100% knock-out score.

The T1-edited plants were selfed to produce T2 plants and crossed to stack all selected mutations into single lines. The *sp* and *sp5g* double mutant was thus selected first, followed by the combination of *sp5g* and *Hygr* mutations. Once the double *sp* and *sp5g* mutant was fixed, it was crossed with the *sp5g* and *HygR* double mutant. The *sp* sp*5g* double mutants were used for the experiments, as the *HygR* mutation does not affect plant architecture (**Supplementary Figure 1**).

### 3.2 Accelerated flowering and compact growth in Tomaffron double mutants

**Figure 3:**
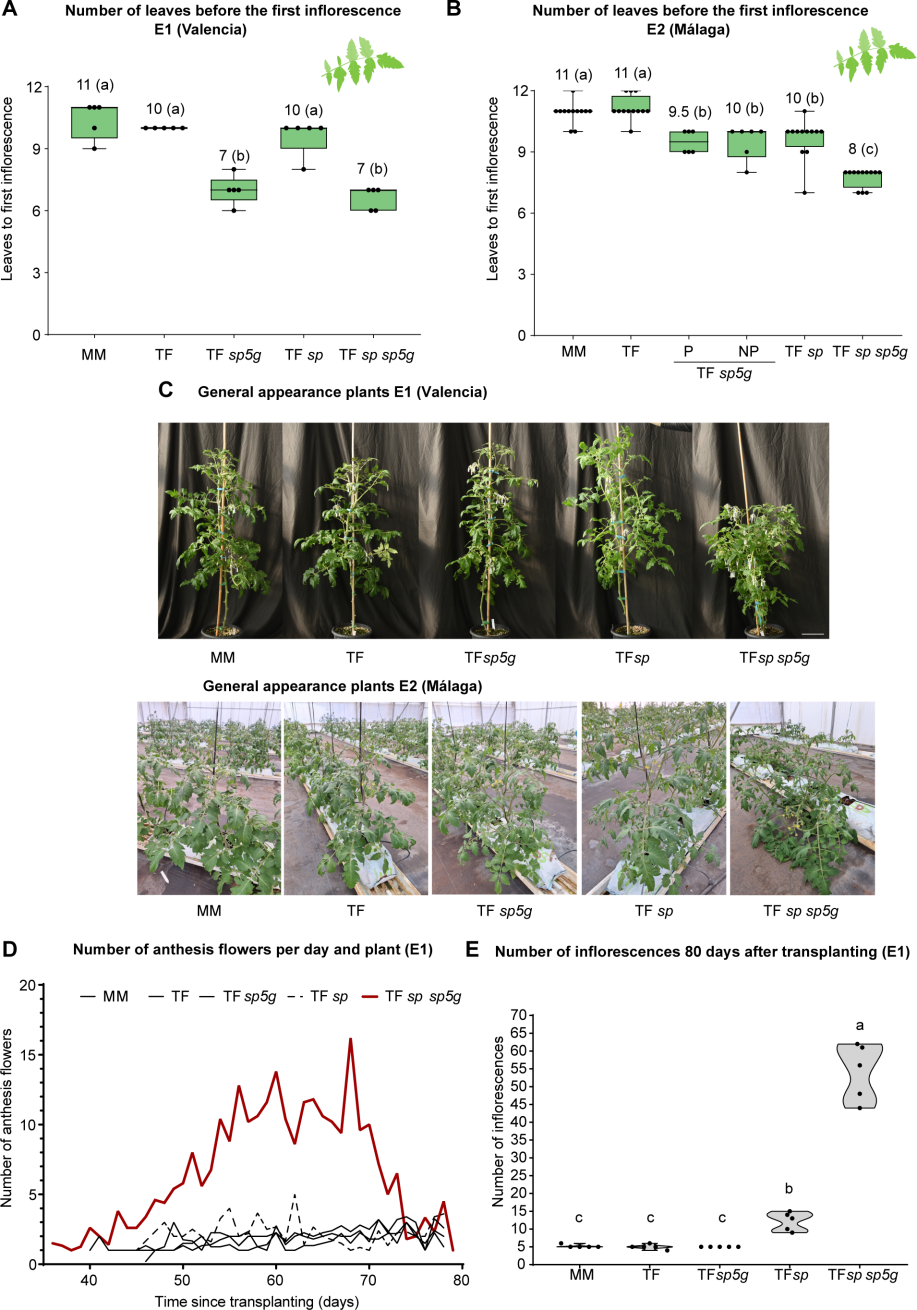
Effect of the *sp* and *sp5g* mutations on flowering time. Number of leaves before the first inflorescence in E1 **(A)** and E2 **(B)** growing sites. **(C)** Plant phenotype in E1 (scale bar: 15 cm) and E2. **(D)** Number of anthesis flowers per day in each plant in E1. Each data point represents the mean of five biological samples. **(E)** Number of inflorescences per plant 80 days after transplanting and plant phenotype in E1. Boxplots represent the median (center line) and interquartile range (25th–75th percentile); whiskers extend from the minimum to the maximum values, and individual data points are shown. Group differences in **(A, B, E)** were assessed using a one-way permutation ANOVA followed by pairwise comparisons (*p*-adj < 0.05). Numbers above the boxplots indicate the median. Different letters indicate statistically significant differences. MM, Moneymaker; TF, Tomaffron; *SP*, self-pruning.

Early flowering and higher productivity are desirable traits in tomato plants. They could be particularly important for our tomato-based crocin biofactory, as it could speed up the process to obtain this metabolite. The double mutant *sp* sp*5g* Tomaffron plants exhibited the earliest flowering phenotypes in Valencia (E1 location) and Málaga (E2 location), followed by the *sp5g*. Early flowering was supported by a reduced number of leaves before the first inflorescence and a shorter time between transplanting and the first anthesis (**Figures 3A, B**; **Supplementary Figure 2**). In the E2 location, *sp5g* mutant plants produced a similar number of leaves before the first inflorescence, regardless of hand-pruning (**Figure 3B**). However, the first anthesis flower appeared four days earlier in hand-pruned plants than in non-pruned plants (**Supplementary Figure 2B**).

Phenotypic differences between the two environments were found with the *sp* mutants. At the E1 location, the Tomaffron *sp* single mutant plants exhibited a delay in the appearance of the first flower compared to both non-mutant and *sp5g* mutant plants (**Supplementary Figure 2A**), but did not show differences in the number of leaves before the first inflorescence compared to non-mutant plants (**Figure 3A**). In contrast, at the E2 location, the *sp* mutants showed flowering timing similar to that of the non-edited and non-pruned *sp5g* plants (**Supplementary Figure 2B**), but a reduced number of leaves before the first inflorescence compared to non-mutant plants (**Figure 3B**). Additionally, at E1, the Tomaffron *sp* mutant plants did not exhibit a compact phenotype, while at E2, the *sp* mutants were determinate (**Figure 3C**). The differing results were not due to variations in the *sp* mutation because all the plants exhibited the same mutation in the *sp* gene (**Supplementary Figure 3**).

To better define the impact of the mutations on agronomic traits, the number of flowers at anthesis per plant was recorded daily at the E1 location, starting from the appearance of the first anthesis flower. In all genotypes, except the double mutant, the average daily number of flowers at anthesis per plant ranged from two to four. In contrast, the double mutant exhibited a 15-day period during which the average daily number of flowers per plant reached at least ten (**Figure 3D**). By 80 days after transplanting, the double mutants had an average of 55 inflorescences per plant, followed by the Tomaffron *sp* plants, which had an average of 12 inflorescences per plant. All other genotypes averaged five inflorescences per plant. These findings align with other phenotypes: the double mutant showed compact, highly branched growth, while Tomaffron *sp* exhibited an intermediate architecture between the compact mutant and the upright habit of pruned plants (**Figures 3C, E**).

### 3.3 Effect of *sp* and *sp5g* mutations on fruit development and ripening

**Figure 4:**
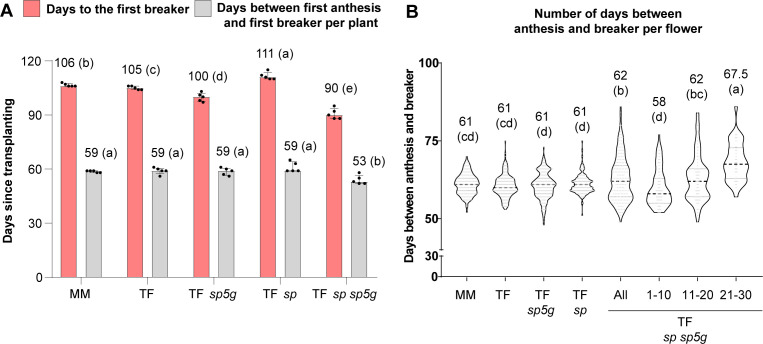
Effect of *sp* and *sp5g* mutations on fruit ripening traits in E1 (Valencia). **(A)** Number of days to the first breaker since transplanting and number of days between the first anthesis and the first breaker. **(B)** Violin plot of the days between anthesis and breaker in all scored flowers from the different genotypes. For the double mutant, total data is represented in the first violin, and then the data are divided into three groups based on the time of the anthesis: early flowers (first 10 days after the first flower in anthesis), middle flowers (from day 11 to day 20), and late flowers (from day 21 to day 30). Bars and violin plots represent the median and interquartile range (25th–75th percentile), showing individual data points. Group differences were assessed using a one-way permutation ANOVA followed by pairwise comparisons (*p*-adj < 0.05). Numbers above the bars and violins indicate the median. Different letters indicate statistically significant differences. MM, Moneymaker; TF, Tomaffron; *SP*, self-pruning.

In Valencia (E1 location), the ripening process of each plant was monitored daily, and the appearance of the first fruit at the breaker stage was recorded. The double mutant plants were the first to produce breaker fruits, with the first fruit appearing 90 days after transplanting, followed by the *sp5g* plants, whose first breaker fruit appeared 10 days later. Fruits of the Tomaffron *sp* plants were the latest to reach the breaker stage, taking five days longer than fruit from MM and Tomaffron control plants (**Figure 4A**).

The days from the anthesis to the breaker stage were scored to assess whether the mutations directly accelerated ripening or only flowering. No significant differences emerged, except in the *sp* sp*5g* double mutant, where ripening occurred six days earlier (**Figure 4A**).

Additionally, the anthesis-to-breaker interval was analyzed over one month. It remained constant across genotypes, except in double mutants, where it was shorter than others in the first 10 days, similar from days 11–20, and longer after day 21 (**Figure 4B**).

### 3.4 The combination of *sp* and *sp5g* mutations resulted in higher yield and earlier fruit production

**Figure 5:**
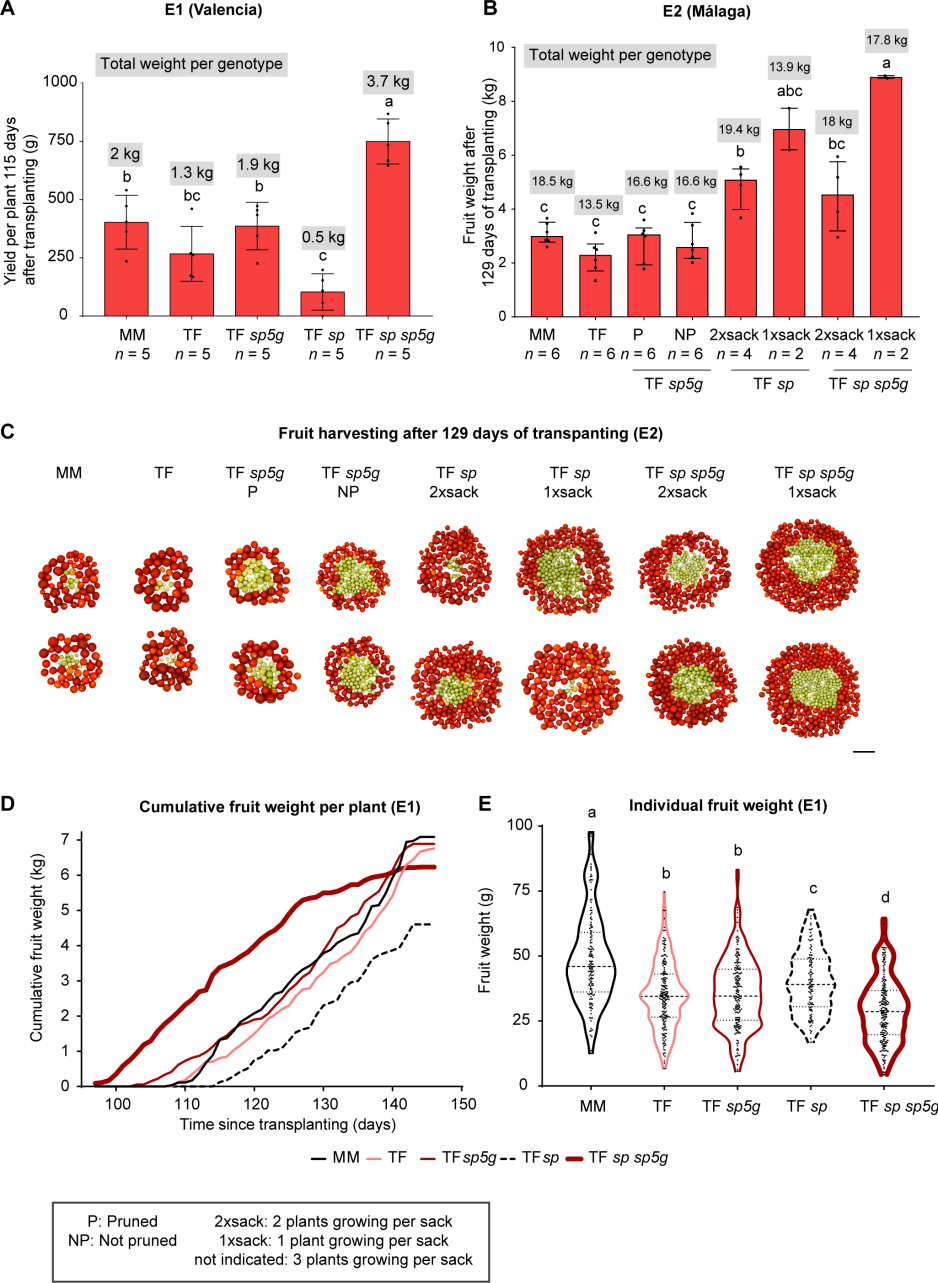
Fruit yield in Tomaffron mutant plants. Yield per plant and genotype 115 and 129 days after transplanting in, respectively, E1 [Valencia, **(A)**] and E2 [Málaga, **(B)**]. **(C)** Pictures after fruit harvesting 129 days after transplanting in E2. Scale bar: 15 cm. **(D)**. Cumulative fruit weight in E1, the weight of each fruit at breaker+10 was scored daily. **(E)**. Violin graph of the individual fruit weight in E1. Bars in **(A)** represent the mean and the standard deviation, showing individual data points. Group differences were determined using ANOVA followed by Tukey’s *post-hoc* test (*p* < 0.05). Bars and violin plots in **(B)** and **(E)** represent the median and interquartile range (25th–75th percentile), showing individual data points. Group differences were assessed using a one-way permutation ANOVA followed by pairwise comparisons (*p*-adj < 0.05). Numbers above the violins indicate the median. Different letters indicate statistically significant differences. MM, Moneymaker; TF, Tomaffron; *SP*, self-pruning.

The Tomaffron *sp* sp*5g* double mutants produced a significantly higher fruit yield per plant than all other genotypes in E1 and E2 when grown without space restrictions. In Valencia (location E1), all red fruits (Br+10 or more) from each plant were harvested 115 days after transplanting. The yield of Tomaffron *sp* mutants was significantly lower than that of other genotypes, except for Tomaffron. MM plants showed a higher yield than Tomaffron, and the *sp5g* single mutants showed an intermediate yield, despite the differences are not significant (**Figure 5A**). In Málaga (location E2), the effect on fruit production and the number of plants per sack could be determined. In E2, all fruits were harvested 129 days after transplanting, and the red fruits were weighed (**Figure 5B**).

Significant differences in red fruit yield were observed in non-hand pruned double mutant plants when grown with (two plants per sack) or without (one plant per sack) growth restrictions. Double mutant plants without growth restrictions achieved the highest red fruit yield, averaging 8.9 kg per plant. Unlike in the E1 location, the second highest yield was obtained from non-restricted Tomaffron *sp* mutants, with an average yield of 7 kg per plant. Growth limitations drastically reduced the yield of these genotypes: in double mutants, the yield was halved, while in *sp* mutants, it was reduced 1.4-fold. Interestingly, no differences in red fruit yield were observed between pruned and non-pruned *sp5g* mutant plants. As in E1, Tomaffron plants showed a non-significant lower yield than MM, though this limitation was partially mitigated by introducing the *sp5g* mutation (**Figures 5B, C**).

In the E1 location, fruit weight production was monitored over time. Tomaffron *sp* sp*5g* double mutants began yielding earlier, with ripe fruits appearing ten days before MM and Tomaffron. Conversely, Tomaffron *sp* mutants exhibited a five-day delay in fruit production compared to these genotypes. By 30 days after the first appearance of ripe fruits, all genotypes, except for Tomaffron *sp*, matched the total fruit weight produced by double mutant plants (**Figure 5D**).

The higher yield of Tomaffron *sp* sp*5g* double mutants in both E1 and E2 was not due to the production of larger fruits but rather to the significantly higher number of smaller fruits compared to single *sp5g* mutants and non-mutant plants (**Figure 5E**; **Supplementary Figure 4**). All this indicates that yield is affected by the growth habit, which is determined by genetic factors, root restriction (e.g., pots or sacks containing different numbers of plants), or manual pruning. In addition, environmental conditions also modulate yield. How these factors influenced crocin production in our Tomaffron biofactory is addressed below.

### 3.5 Early yield in *sp* sp*5g* double mutants results in more compact plants and precocious crocin production

Crocin accumulation was evaluated in each genotype. Tomaffron showed a similar crocin profile in both environments, and in Málaga (E2), no significant differences in crocin concentration in ripe fruit were observed between Tomaffron and any mutant lines. However, in Valencia (E1), Tomaffron *sp* and the double mutant showed significantly higher crocin levels than Tomaffron and Tomaffron *sp5g* (**Supplementary Figure 5**).

**Figure 6:**
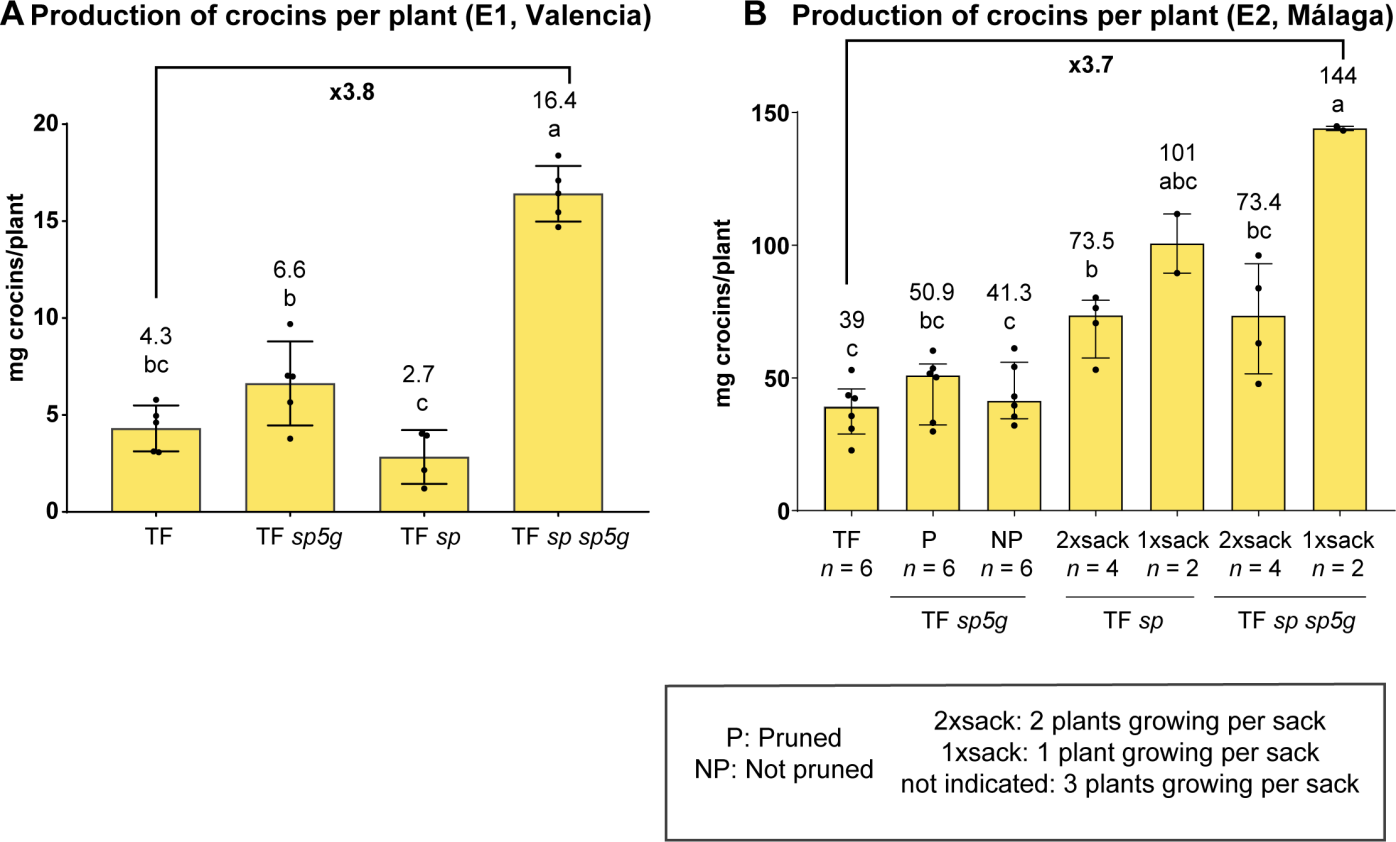
Crocin production per plant at harvest in Valencia (E1) **(A)** and Málaga (E2) **(B)**. Bars in **(A)** represent the mean and the standard deviation, showing individual data points. Group differences were determined using ANOVA followed by Tukey’s *post-hoc* test (*p* < 0.05). Bars in **(B)** represent the median and interquartile range (25th–75th percentile), showing individual data points. Group differences were assessed using a one-way permutation ANOVA followed by pairwise comparisons (*p*-adj < 0.05). Numbers above the bars indicate the mean **(A)** or the median **(B)**. Different letters indicate statistically significant differences. MM, Moneymaker; TF, Tomaffron; *SP*, self-pruning.

Crocin accumulation in fruit was combined with the yield per plant at harvest (115 days after transplanting in E1 and 129 days in E2 locations) to estimate total crocin production per plant. The double mutant plants produced significantly higher amounts of crocins per plant in both environments. In E1 and E2 under non-restricted growth conditions, it was 3.8 and 3.7 times higher than in Tomaffron, respectively. In E2, with two plants growing per sack, it was 1.9 times higher. In E1, pruned *sp5g* mutants produced 1.4 times more crocin than non-edited Tomaffron. In E2, pruned and non-pruned *sp5g* plants showed 1.3- and 1.1-fold increases, respectively (**Figure 6**).

Differences in crocin production related to the *sp* mutation between environments were also observed. In E1, *sp* mutant plants produced half the amount of crocins compared to the non-edited Tomaffron. Conversely, in E2, *sp* mutant plants showed high crocin accumulation levels in high plant density conditions. They exhibited a 2.6-fold increase under non-restricted conditions of lower plant density and a 1.9-fold increase with higher plant density (**Figure 6**).

The combination of the *sp* and *sp5g* mutations consistently quadrupled total crocin production in Tomaffron plants across both environments, demonstrating their synergistic effect. In contrast, the *sp* mutation alone increased crocin production only in E2. These findings highlight the potential of editing the two genes to achieve earlier and enhanced crocin yields in Tomaffron plants. Furthermore, the *sp* and *sp5g* mutations were successfully combined with the *hygr* #6 mutation, generating plants with accelerated crocin production and no selectable marker gene remaining from the transformation process.

### 3.6 Changes in fruit firmness in Tomaffron double mutant plants

**Figure 7:**
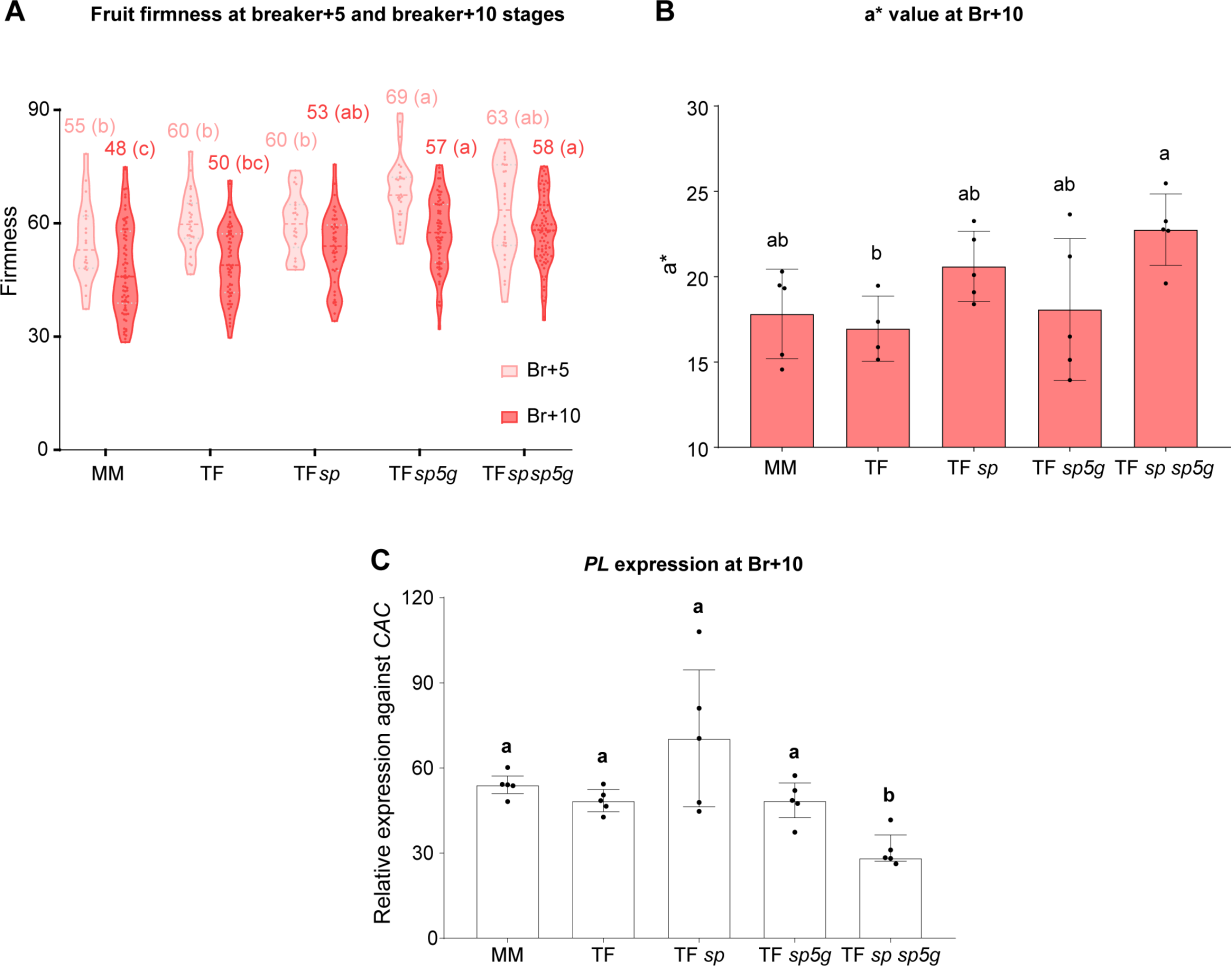
Effect of the *sp* and *sp5g* mutations on fruit firmness in Tomaffron **(A)** Violin plot of firmness of fruits at breaker+5 and breaker+10 stages. Each point represents the mean of two technical replicates for each biological replicate. **(B)** Colorimetric a* value of fruits at breaker+10. **(C)** Gene expression of pectate lyase at breaker+10. Bars in **(A, B)** represent the mean and the standard deviation, showing individual data points. Group differences in **(A)** were assessed using Welch’s one-way ANOVA followed by Games–Howell *post hoc* comparisons. Group differences in **(B)** were determined using ANOVA followed by Tukey’s *post-hoc* test (*p* < 0.05). Bars in **(C)** represent the median and interquartile range (25th–75th percentile), showing individual data points. Group differences were assessed using a one-way permutation ANOVA followed by pairwise comparisons (*p*-adj < 0.05). Numbers above the bars and violins indicate the mean **(A, B)** or the median **(C)**. Different letters indicate statistically significant differences. MM, Moneymaker; TF, Tomaffron; *SP*, self-pruning.

Maintaining a moderate level of firmness during ripening is a key trait in tomatoes for both the fresh and processing markets, particularly for the processing market, as good firmness at the red ripe stage ensures that fruits remain sound on the plant, allowing for one-time mechanical harvesting. Fruit firmness was evaluated at two ripening stages: 5 (light red) and 10 (red) days after the breaker stage. Most genotypes analyzed showed fruits with similar firmness values except for the plants carrying the *sp5g* mutation, either alone or combined with the *sp* mutations, which produced fruits with higher firmness at both stages (**Figure 7A**).

To assess whether increased firmness in *sp5g* plants resulted from delayed ripening, fruits were harvested at Br+3, and CIELab color parameters were measured until Br+10. No clustering appeared early, but after Br+5, *sp* sp*5g* and *sp5g* fruits diverged from other genotypes (**Supplementary Figures 6**, **7**). At Br+10, *sp* sp*5g* fruits had significantly higher a* values than non-edited Tomaffron, indicating redder coloration (**Figure 7B**). Since *sp* and *sp5g* single mutants showed no significant differences, this suggests an effect of both mutations combined. These results confirm that the higher firmness values in *sp5g* mutants are not associated with delayed ripening.

To elucidate the molecular changes occurring during the ripening process, we evaluated the expression of the pectate lyase (*PL*) and polygalacturonase 2A (*PGA2*) genes, involved in cell wall remodeling, as well as key regulators of fruit carotenoid biosynthesis and ripening, phytoene synthase 1 (*PSY1*), the ripening inhibitor (*RIN*) and 1-aminocyclopropane-1-carboxylate synthase (*ACS2*) genes. Only the double mutant plants showed a statistically significant decrease in *PL* expression (**Figure 7C**; **Supplementary Figure 8**).

## 4 Discussion

### 4.1 Genome editing is the best approach to introduce the *sp* and *sp5g* mutations in Tomaffron

The natural *sp* mutation in the *SELF-PRUNING* gene has been extensively used to obtain determinate tomato plants and was a revolutionary advancement that facilitated mechanical harvesting for processing tomato (Eshed and Lippman, 2019). Notable examples include the introduction of the *sp* mutation, together with the compound inflorescence mutation, by crossing in a breeding program to the Penjar tomato, cultivated in the Mediterranean basin (Schober et al., 2022), and the combination of the *sp* and *dwarf* mutations in Salad-type tomato lines, resulting in hybrid plants that exhibited higher yields compared to the parental lines and are being used to obtain homozygous lines in subsequent generations (Pereira et al., 2024). A transgenic tomato line accumulating the miraculin glycoprotein from *Richadella dulcifica* fruit in a MM background was crossed with Micro-Tom to introgress the *sp* mutation and produce higher amounts of this sweetener per square meter. The production of miraculin per year and square meter increased eightfold with the introgression of the *sp* mutation. However, it took five generations to obtain the plants with the desired traits (Kato et al., 2010).

The advantage of introducing *sp* by crossing is that beneficial variability in other traits can be introduced from the donor plant alongside the mutation. However, it may not be the fastest method if the sole target is the *SP* gene. In tomato Micro-Tom plants, the *glutamate decarboxylase* (*GAD*) gene was targeted using CRISPR-Cas9, and edited plants with up to 15 times more γ-aminobutyric acid (GABA) were obtained (Nonaka et al., 2017). These plants were crossed with the “Aichi First” tomato cultivar, and the F1 plants already showed higher levels of GABA (Lee et al., 2018). One fixed line from this cross was used to edit the *SP* and *DWARF* genes, and the desired traits were obtained in the T1 generation, maintaining the preferred traits from the original variety (Nagamine and Ezura, 2024). In the present study, *SP, SP5G*, and the hygromycin resistance genes were targeted independently to elucidate the effect of each mutation. Additionally, a construct targeting the three genes simultaneously was prepared for future experiments requiring a faster approach.

An additional disadvantage of conventional breeding approaches is the possibility that the mutation of interest is linked to an undesired trait. Recently, Tomaffron was crossed with Xantomato, a quadruple mutant tomato plant that accumulates high levels of β-carotene and zeaxanthin in the fruit (Karniel et al., 2020; Lobato-Gómez et al., 2024). The substrate of *Cs*CCD2 is zeaxanthin, and the resulting transgenic plants containing the mutations accumulated higher levels of saffron apocarotenoids. It was found that the crucial mutation for obtaining the highest levels of these valuable metabolites was the *BETA* mutation from *Solanum habrochaites* (*B^Sh^*) (Lobato-Gómez et al., 2024). *BETA* encodes for a lycopene β-cyclase, and *B^Sh^* mutant plants exhibit a significantly higher gene expression than wild-type plants and accumulate high levels of β-carotene instead of lycopene in ripe fruit (Ronen et al., 2000). The *BETA* gene has been mapped close to the *SP* locus to chromosome 6, and all cultivars that showed a determinate phenotype also showed red fruits and, consequently, the non-mutant *BETA* allele (Pnueli et al., 1998).

The linkage of wild-type *BETA* with the *sp* mutant allele makes it very unlikely to find a tomato line with determinate growth that can be used to obtain high levels of saffron apocarotenoids. Moreover, crossing indeterminate *CsCCD2* transgenic MM lines and *B^Sh^* mutant plants with a determinate cultivar would make segregating the wild-type *BETA* allele from the *sp* mutation difficult.

### 4.2 The Tomaffron *sp* mutant behaves differently in the evaluated environments

Tomaffron *sp* mutants exhibited some differences in results across the two environments. In E1, the mutants showed delayed flowering (**Supplementary Figure 2A**) and ripening (**Figure 4A**), which led to a lower yield in fruit and crocin production at harvest (**Figures 5A, D**, **6A**). Additionally, they did not display a compact phenotype (**Figure 3C**). In contrast, in E2, Tomaffron *sp* mutants showed the same flowering time (**Supplementary Figure 2B**) and a significantly lower number of leaves before the first inflorescence compared to non-mutant plants (**Figure 3B**), together with a significantly higher yield than non-edited Tomaffron and TF *sp5g* single mutant when grown under space restricted conditions (**Figures 5B, C**).

The results in E1 were unexpected, as the single mutation of *sp* targeted by CRISPR/Cas9 resulted in a determinate phenotype in *Solanum pimpinellifolium* (Zsögön et al., 2018). Other studies have introduced the *sp* mutation into indeterminate cultivars, but this was achieved through crossing, during which other traits associated with compactness were selected alongside the *sp* mutation, resulting in the absence of single *sp* mutants (Schober et al., 2022; Pereira et al., 2024). The *sp* mutation has been introduced into MM by crossing a transgenic MM line over-producer of miraculin with Micro-Tom, and, as in other studies, it was selected together with the *dwarf* mutation, preventing the ability to study the effect of single *sp* mutants on plant architecture and flowering (Kato et al., 2010). This is the first report of a single *sp* mutation introduced by CRISPR/Cas9 into MM.

The sequencing results shown that the plants grown in both environments had the same *sp* mutation (**Supplementary Figure 3**), suggesting that the growing conditions influence the determination of the Tomaffron *sp* mutants. In both environments, Tomaffron *sp* plants were not subjected to hand pruning. However, in E1, the plants exhibited upright growth rather than a compact habit and did not show a determinate growth pattern (**Figure 3C**). In E1, plants were grown at a very high density (11.11 plants/m^2^) in pots, whereas in E2, they were grown at a lower density (0.53 or 1.066 plants/m^2^) in sacks, and this difference in density appears to have affected plant architecture in *sp* mutants but not in the double *sp* sp*5g* mutants. Our results indicate that the differences observed in Tomaffron *sp* in both environments are not due to genetic variation but rather to environmental and growth conditions. In addition to differences in plant spacing and growth systems between the two greenhouses, temperature control also differed, and the plants were grown at different times of the year. A more in-depth study manipulating specific environmental and growth parameters is needed to elucidate the causes of the different behavior observed in Tomaffron *sp* mutants across the two environments evaluated in this study.

Although the effect of the single *sp* mutation in E1 was not as expected, it impacted the phenotypes assessed when combined with the *sp5g* mutation. The *sp* sp*5g* mutant plants exhibit a compact phenotype and accelerated flowering and yield compared to the single *sp5g* mutant plants, as previously demonstrated (Soyk et al., 2017).

### 4.3 Effect on the spacing of the plants in *sp* and double *sp* sp*5g* Tomaffron mutants

In the E1 experiment, all genotypes were grown at the same density, and the Tomaffron double mutant showed a clear increase in fruit and crocin yield compared to the other genotypes (**Figures 5A**, **6A**). A different approach was applied in the E2 experiment, where we aimed to evaluate the effect of plant spacing on the yield of Tomaffron single *sp* and double *sp* sp*5g* mutants. These two genotypes were grown at two different plant densities: 1.066 and 0.53 plants/m^2^.

Our results showed that fruit and crocin production per plant were higher with increased spacing for both genotypes, but statistically different only in the double mutants (**Figures 5B**, **6B**). Proper spacing enhances the exposure of tomato plants to light, promoting nutrient accumulation and improving fruit quality (Wamser et al., 2012). However, the yield per unit area does not correlate with the yield per plant due to the additional space required to grow the plants. It was demonstrated that the total yield of double mutant plants increased significantly by planting them at higher densities (Soyk et al., 2017), and this is a promising strategy to increase even further the production of crocins per square meter.

In the Tomaffron double mutant, increased productivity per plant offsets the greater space requirement, resulting in similar fruit yields at both densities. In contrast, for Tomaffron *sp*, the negative effect of increased spacing outweighed the higher yield per plant, leading to a 1.4-fold decrease in total yield at lower density (**Figure 5B**). Similar findings have been reported in other determinate (Balemi, 2008; Çetin and Uygan, 2008; Dipple et al., 2022) and indeterminate (Balemi, 2008; Nkansah et al., 2021) tomato cultivars, where increased spacing did not boost yield per unit area.

### 4.4 Comparison of the production per square meter in Tomaffron double mutant and *Crocus sativus*

Saffron yield can be measured in various ways, including the fresh or dry weight of saffron flowers or the yield of dried stigmas (Ghanbari et al., 2019; Tashakkori et al., 2021). Producing one kilogram of saffron spice requires nearly 80 kilograms of flowers (Shahi et al., 2016), and crocins constitute up to 10% of saffron stigma (Demurtas et al., 2019). In Iran, the leading saffron producer, the average saffron yield is 3.96 g/m^2^, with a maximum yield of approximately 7.5 g/m^2^ (Farrokhi et al., 2021), translating to up to 396 mg/m^2^ of crocins.

In E1, where all plants were grown at the same density, the production per square meter of the different genotypes was equivalent to the production per plant. With a very high plant density of 11.11 plants/m^2^, Tomaffron produced 47.8 ± 13.1 mg crocins/m², while the double *sp* sp*5g* mutant Tomaffron yielded 182.1 ± 15.9 mg crocins/m^2^ (**Table 1**). Although the production of crocins per square meter remains higher in *C. sativus*, saffron is the most expensive spice due to its labor-intensive manual harvesting process, despite efforts to partially automate it (Saeidirad, 2020; Denarda et al., 2021). To match the average crocin yield per square meter in Iran, the fruits from approximately 20 Tomaffron *sp* sp*5g* plants grown in high-density would need to be harvested (**Figure 5E**; **Table 1**). Unlike saffron plants, tomatoes can be harvested mechanically, and the compact phenotype displayed by the mutated plants facilitates mechanization. Furthermore, the harvested tomatoes can be processed into products that are efficient for storing saffron apocarotenoids for a prolonged time (Lobato-Gómez et al., 2025). The production of these plants could be further enhanced by using *B^Sh^* mutant plants expressing *Cs*CCD2 (Lobato-Gómez et al., 2024) as the recipient plants for the transformation with the CRISPR/Cas9 cassette designed to target the three *sp*, *sp5g*, and *HygR* genes. This approach could potentially achieve a crocin yield of 1 gram per square meter.

**Table 1:** Crocin yield in both environments.

E1 (Valencia)					
Genotype	N of replicates	Density (plants/m^2^)	Fruit yield per plant (g)	Crocin production (mg/plant)	Crocin yield (mg/m^2^)
MM	5	11.11	402.8 ± 115.8		
TF	5		266.8 ± 118.1	4.31 ± 1.18	47.81 ± 13.14
TF *sp5g*	5		386.3 ± 102.1	6.62 ± 2.16	73.53 ± 24.02
TF *sp*	5		103.4 ± 78.1	2.74 ± 1.21	24.32 ± 17.89
TF *sp* sp*5g*	5		749.4 ± 96.7	16.41 ± 3.69	182.15 ± 15.91
E2 (Málaga)					
Genotype	N of replicates	Density (plants/m^2^)	Fruit yield per plant (kg)	Crocin production (mg/plant)	Crocin yield (mg/m^2^)
MM	6	1.6	3.09 ± 0.41		
TF	6		2.24 ± 0.63	38.01 ± 10.60	60.81 ± 16.97
TF *sp5g* P	6		2.77 ± 0.73	46.45 ± 12.14	74.32 ± 19.42
TF *sp5g* NP	6		2.78 ± 0.71	44.26 ± 11.25	70.82 ± 18.00
TF *sp* 2xsack	4	1.066	4.85 ± 0.83	70.08 ± 11.99	74.70 ± 12.78
TF *sp* 1xsack	2	0.53	6.97 ± 1.09	100.60 ± 15.74	53.34 ± 8.34
TF *sp* sp*5g* 2xsack	4	1.066	4.50 ± 1.33	72.69 ± 21.51	77.49 ± 22.93
TF *sp* sp*5g* 1xsack	2	0.53	8.91 ± 0.69	144.00 ± 1.11	76.32 ± 0.59

Data is presented as mean ± standard deviation.P, pruned; NP, non-pruned. In E2, 2xsack (2 plants growing per sack); 1xsack (1 plant growing per sack); not indicated (3 plants growing per sack); MM, Moneymaker; TF, Tomaffron; *SP*, self-pruning.

Tomato has advantages such as wide cultivation, adaptability to open and greenhouse conditions, and a short life cycle, with ripe fruits ready 3–4 months after transplanting (Li et al., 2018). In contrast, saffron is harvested 4–6 months after planting, with low initial yields due to small corms, but productivity increases in subsequent generations as new corms form and existing ones gain weight (McGimpsey et al., 1997; Lage and Cantrell, 2009).

The higher crocin content in Tomaffron *sp sp5g* mutants, along with the absence of a significant increase in crocin content in E2, and a slight but significant increase in E1 indicates that the double mutant plants produce more crocins due to changes in plant architecture rather than other effects of the mutations on crocin accumulation. Apart from the higher fruit and crocin production yields, the Tomaffron *sp sp5g* plants produced fruits with greater firmness. These fruits did not exhibit delayed ripening but instead exhibited significantly lower gene expression of the pectate lyase (**Figure 7C**), responsible for pectin degradation, the primary component of the tomato fruit cell wall (Yang et al., 2017). A recent study demonstrated that the CRISPR/Cas9 knock-out of pectate lyase results in increased fruit firmness (Fumelli et al., 2025), suggesting that the lower expression of this gene in our edited lines may be responsible for the higher firmness observed in Tomaffron *sp sp5g* fruits, although the cause of this reduced expression was not determined in the present study.

Taken together, the results of this study demonstrate that Tomaffron *sp sp5g* double mutant plants are an excellent platform for producing high levels of saffron apocarotenoids while having a growth habit that facilitates early and mechanical harvest of the fruits. The crocin production per square meter is almost fourfold lower than that of *C. sativus*, but it requires significantly less effort to harvest and process the material. Optimization of crocin yield per square meter requires not only editing *sp* and *sp5g* but also the appropriate conditions, like plant density, and other environmental conditions that clearly affect the final phenotypes. Future experiments could focus on producing double mutants of transgenic plants that accumulate higher levels of saffron apocarotenoids or introducing additional mutations to incorporate other traits demanded by the processing tomato industry.

## Data Availability

The datasets presented in this study can be found in online repositories. The names of the repository/repositories and accession number(s) can be found below: https://doi.org/10.6084/m9.figshare.6025748.
