# Supplementary material for: Optimizing a tomato crocin biofactory by fine-tuning plant architecture
Source: Front Plant Sci. 2026 Jan 21;16:1730399. doi: 10.3389/fpls.2025.1730399 (PMC12868218; doi:10.3389/fpls.2025.1730399)
Supplement: Supplementary file 1 [file DataSheet1.docx]

Supplementary Material

# Supplementary Tables

**Supplementary Table 1**. Guide RNAs (gRNAs) used to target each of the genes with *Sp*Cas9 and the primers used to genotype by PCR followed by Sanger sequencing. *SP*: self-pruning. *HygR*: hygromycin resistance gene.

|  | Strand | gRNA + **PAM** sequence | Primers for genotyping | |
| --- | --- | --- | --- | --- |
| *Hygromycin resistance* (*HygR*) | | | | |
| PNOS-1 | + | TATGACCCCCGCCGATGACG**CGG​** | FW | TCATTAGGGACTCCTGACGGA |
| PNOS-2 | + | ATGACCCCCGCCGATGACGC**GGG** | RV | CCAAAATCCAGTGACCTCGC |
| HygR-1 | - | **CCG**TGGTTGGCTTGTATGGAGCA |  | |
| HygR-2 | - | **CCG**CATTGGTCTTGACCAACTCT |  |  |
| TNOS-1 | + | ACTTTTCGGGGAAATGTGCG**CGG** |  |  |
| TNOS-2 | - | **CCG**CTCATGAGACAATAACCCTG​ |  |  |
|  |  |  |  | |
| *Self-pruning* (*SP*) Solyc06g074350 | | |  |  |
| SP-1 | - | **CCT**TCCTCAGTAACTTCTAAACC | FW | TGATTGGTAGAGTGATTGGTGAAG |
| SP-2 | + | TCTCAGATCCTTCTTCACAC**TGG** | RV | GTGTAGATGTTCCCTGAGATATGG |
|  | | |  |  |
| *Self-pruning 5G* (*SP5G*) Solyc05g053850 | | |  |  |
| SP5G-1 | + | AAGATGTGTAGACTTTGGTG**TGG** | FW | CATCCATCCATCTCATGTAATAAAC |
| SP5G-2 | - | **CCT**TGAGGCCTTCACAAGTTGTC | RV | GCCTTTGCACATATTGTCCT |

**Supplementary Table 2**. Primers used for the gene expression analyses.

| Gene | Forward primer | Reverse primer |
| --- | --- | --- |
| CLATHRIN ADAPTOR COMPLEXES SUBUNIT (*CAC*) | AAGGTGTGGGAGAAGAGTGG | ATTGCATCCCTCAAATAGGGC |
| Polygalacturonase 2A (*PGA2*) | TCTCAAAATGTGCAGGCCAC | CCTAACTCCATTTTCGGCACC |
| Ripening Inhibitor (MADS-box) (*RIN)* | ATCATGGCATTGTGGTGAGC | TGATGGTGCTGCATTTTCGG |
| Pectate lyase (*PL*) | AGCTTCGAGTTTGAGTGCAAG | ATCCCCTTTTGCTTTGGTTCT |
| Phytoene synthase 1 (*PSY1*) | TGTGACGTCTCAAATGGGACAA | GTCATCGTCCGTTCTCCAGAT |
| 1-aminocyclopropane-1-carboxylate synthase (*ACS2*) | CGAGGATTCGGAGGTTCGTA | AGGTGACGAAAGTGGTGACA |

**Supplementary Table 3**. Percentage of insertion and/or deletions (indel) and knock-out score in each of the putative edited lines. The knock-out score refers to indel mutations that result in a frameshift, excluding indels multiples of 3 until 21 bp. Only the indels that showed a percentage higher than 10% are represented in the table. The lines indicated in bold are the ones selected for further experiments. *SP*: self-pruning. *HygR*: hygromycin resistance gene.

| Targeted gene | Line | Indel % | Knockout-Score % | Indel (above 10%) |
| --- | --- | --- | --- | --- |
| *SP* | #1A | 100 | 99 | +1 (51%), -4 (16%), -28 (16%) |
|  | #1C | 100 | 100 | -57 (100%) |
|  | #2 | 77 | 77 | +1 (26%), 0 (23%), -11 (18%), +2 (13%), -7 (11%) |
|  | **#4** | **100** | **100** | **+2 (22%), +2 (20%), +1 (50%)** |
|  | #5B | 100 | 89 | -7 (38%), -1 (39%), -6 (11%) |
|  | #20 | 100 | 100 | +1 (60%), -11 (21%) |
|  | #23 | 100 | 100 | +1 (54%), +2 (22%), +2 (19%) |
|  | #24 | 100 | 79 | -4 (31%), +1 (27%), +2 (17%), -3 (21%) |
| *SP5G* | #1B | 100 | 98 | -45 (57%), -1 (23%) |
|  | #4A | 100 | 93 | -45 (29%), -59 (27%), -5 (24%) |
|  | **#6** | **100** | **100** | **-5 (82%)** |
| *HygR* | **#6B** | **100** | **100** | **-617 (100%)** |
|  | #1 | 30 | 30 | 0 (70%), -2 (14%), -1 (14%) |

## Supplementary Figures


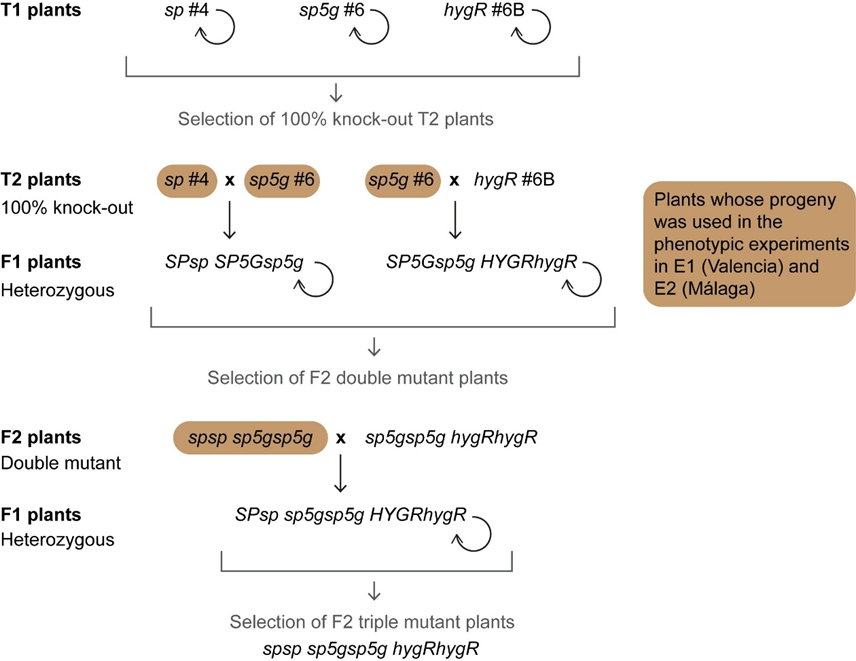


**Supplementary Figure 1**. Schematic representation of the crosses performed to obtain the double and triple mutant plants. *SP*: self-pruning. *HygR*: hygromycin resistance gene.


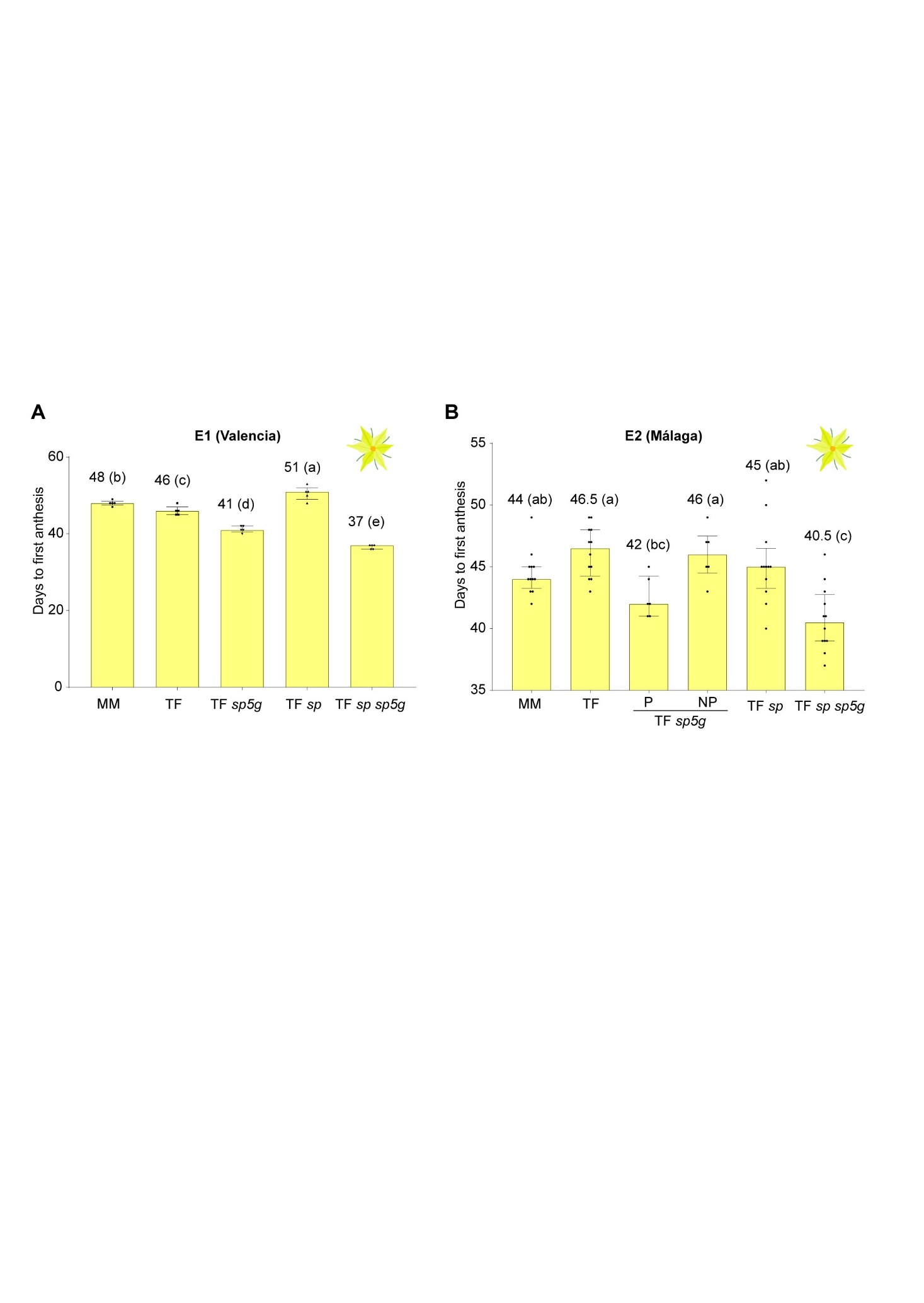


**Supplementary Figure 2**. Number of days to the first inflorescence in E1 (**A**) and E2 (**B**). Bars represent the median and interquartile range (25th–75th percentile), showing individual data points. Group differences were assessed using a one-way permutation ANOVA followed by pairwise comparisons (*p*-adj < 0.05). Numbers above the bars indicate the median. Different letters indicate statistically significant differences. MM: Moneymaker; TF: Tomaffron. *SP*: self-pruning. P: pruned. NP: not pruned.


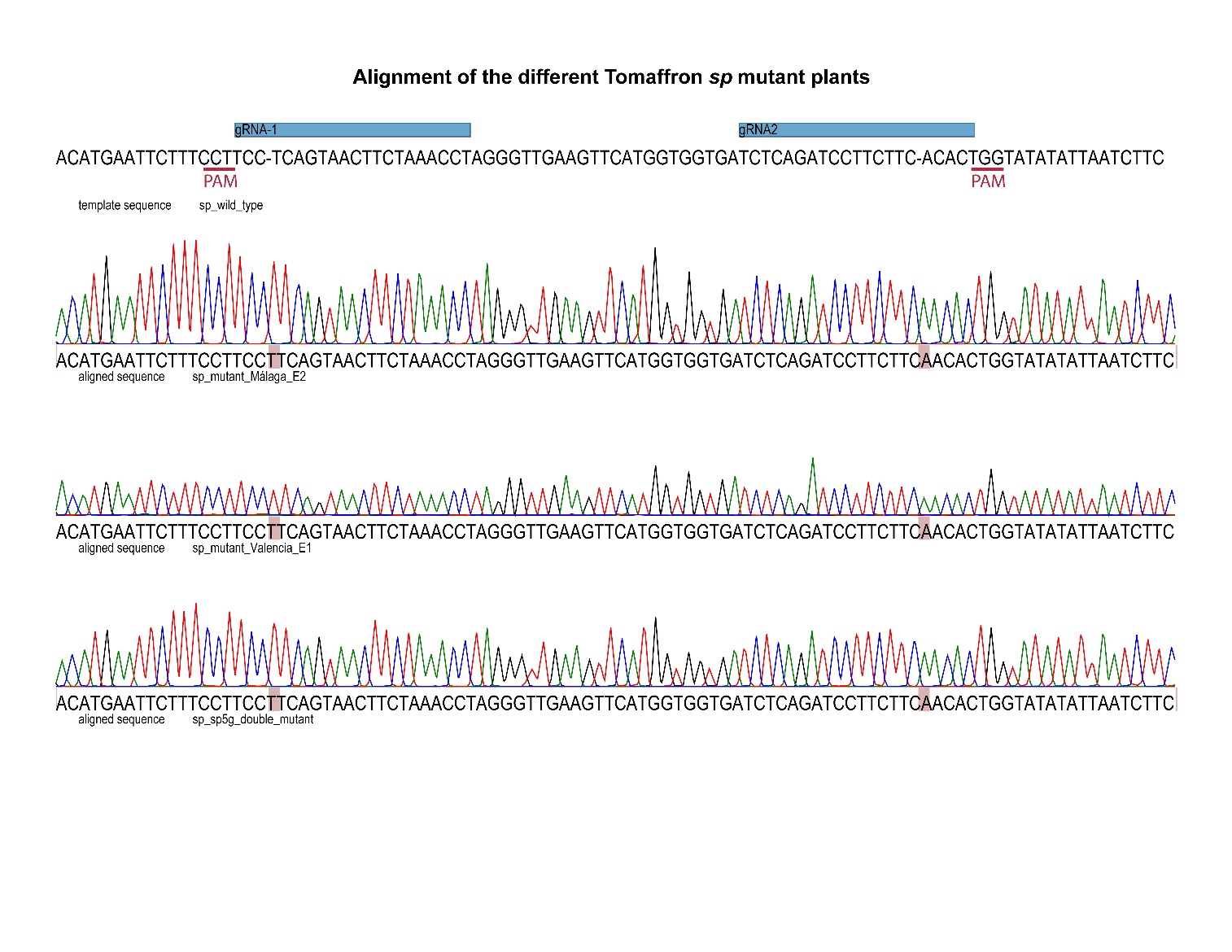


**Supplementary Figure 3**. Chromatogram of Tomaffron *sp* mutants aligned with the wild-type *SP* gene from Tomaffron. *SP*: self-pruning.


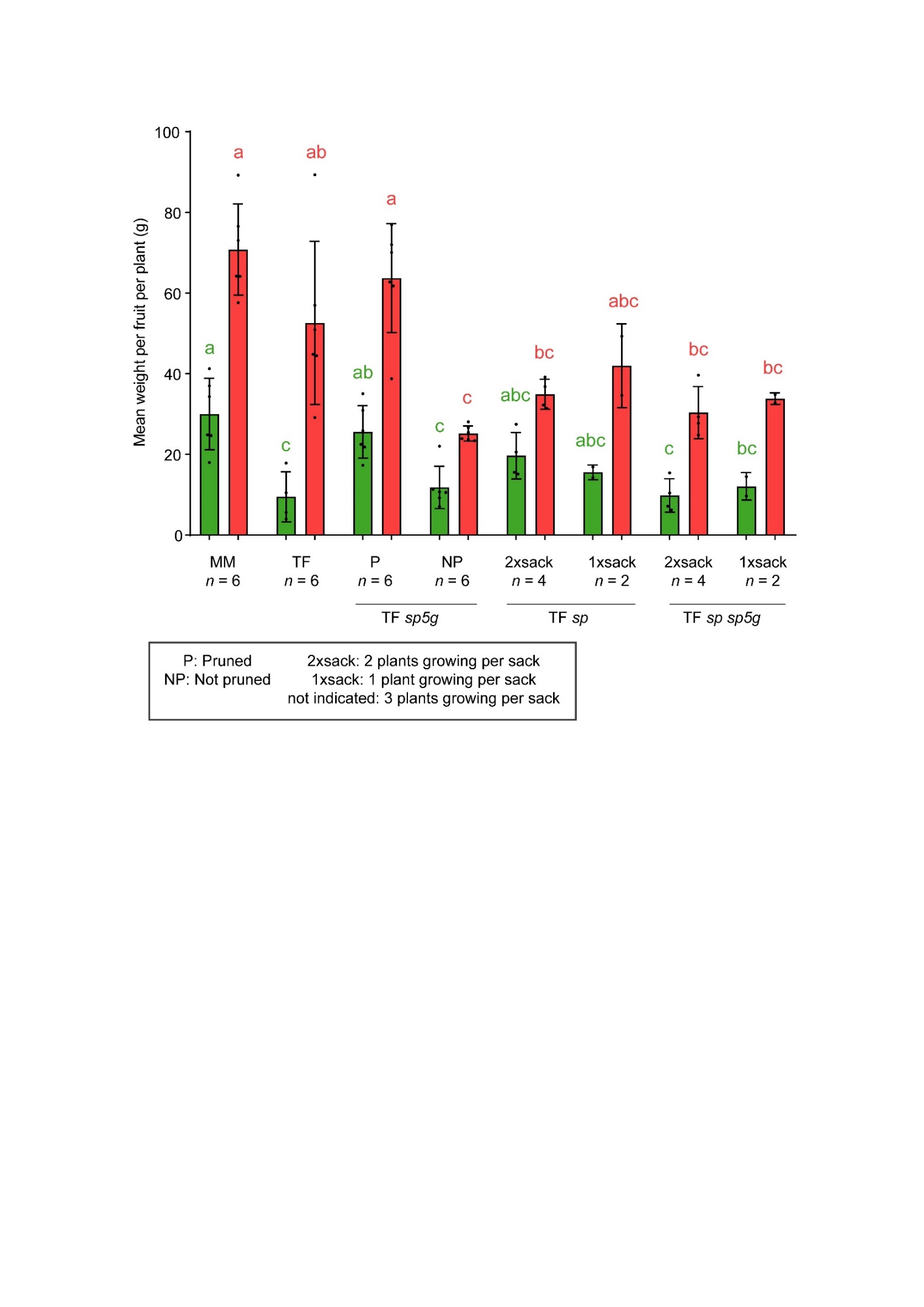


**Supplementary Figure 4**. Mean fruit yield per plant, divided by the number of fruits for each genotype. Bars represent the median and interquartile range (25th–75th percentile), showing individual data points. Group differences were assessed using a one-way permutation ANOVA followed by pairwise comparisons (*p*-adj < 0.05). Different letters indicate statistically significant differences. MM: Moneymaker; TF: Tomaffron. *SP*: self-pruning.


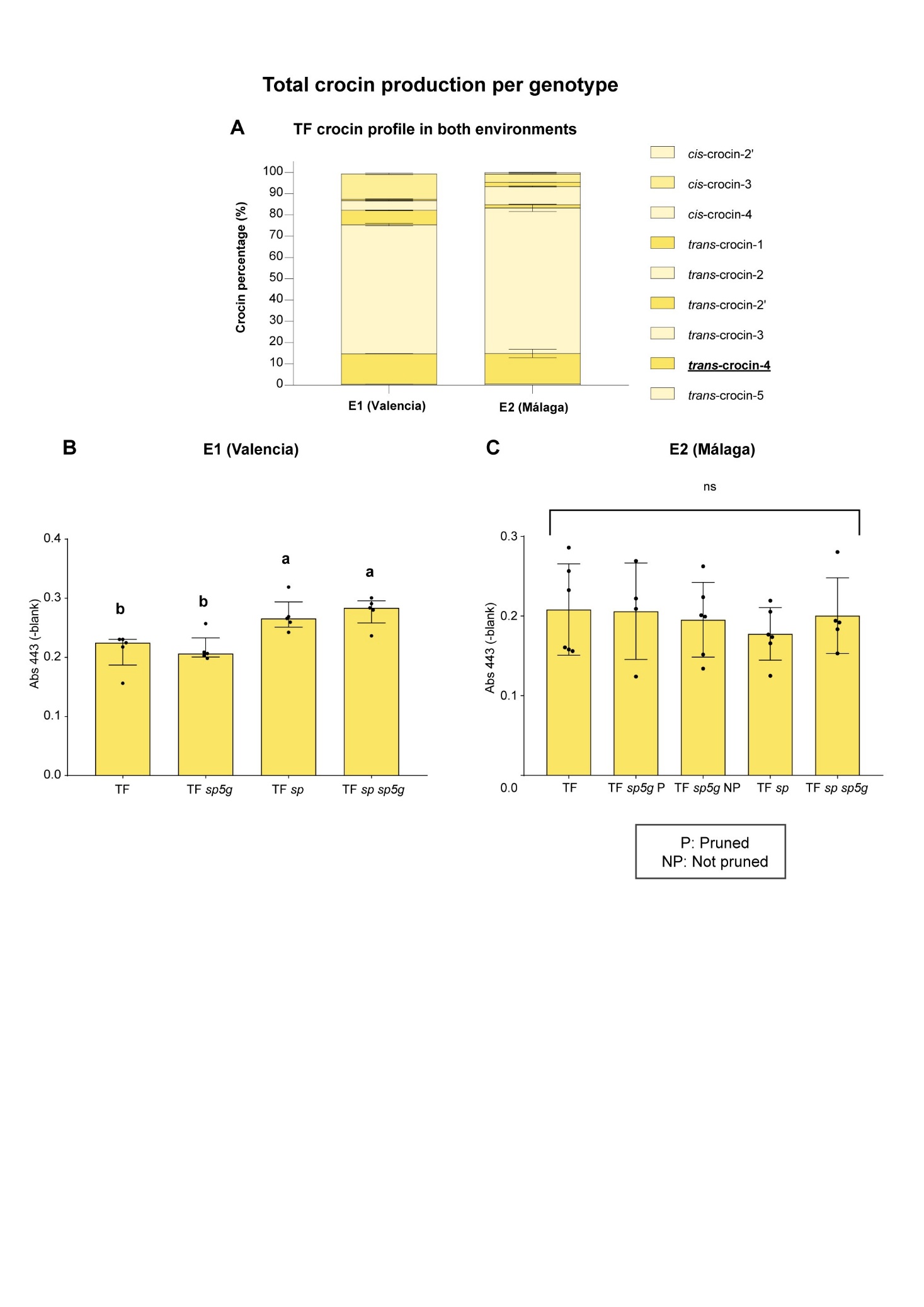


**Supplementary Figure 5**. **A**. Crocin profile of TF determined by LC-MS. Total crocin production in Valencia (E1, **B**) and Málaga (E2, **C**). Each dot represents the mean of three technical replicates for each biological replicate (*n* ≥ 4). Bars in **A** represent the median and interquartile range (25th–75th percentile), showing individual data points. Group differences were determined using a one-way permutation ANOVA followed by pairwise comparisons (*p*-adj < 0.05). Bars in **B** represent the mean and the standard deviation, showing individual data points. Group differences were assessed using ANOVA followed by Tukey’s post-hoc test (*p* < 0.05). Different letters indicate statistically significant differences. MM: Moneymaker; TF: Tomaffron. *SP*: self-pruning.


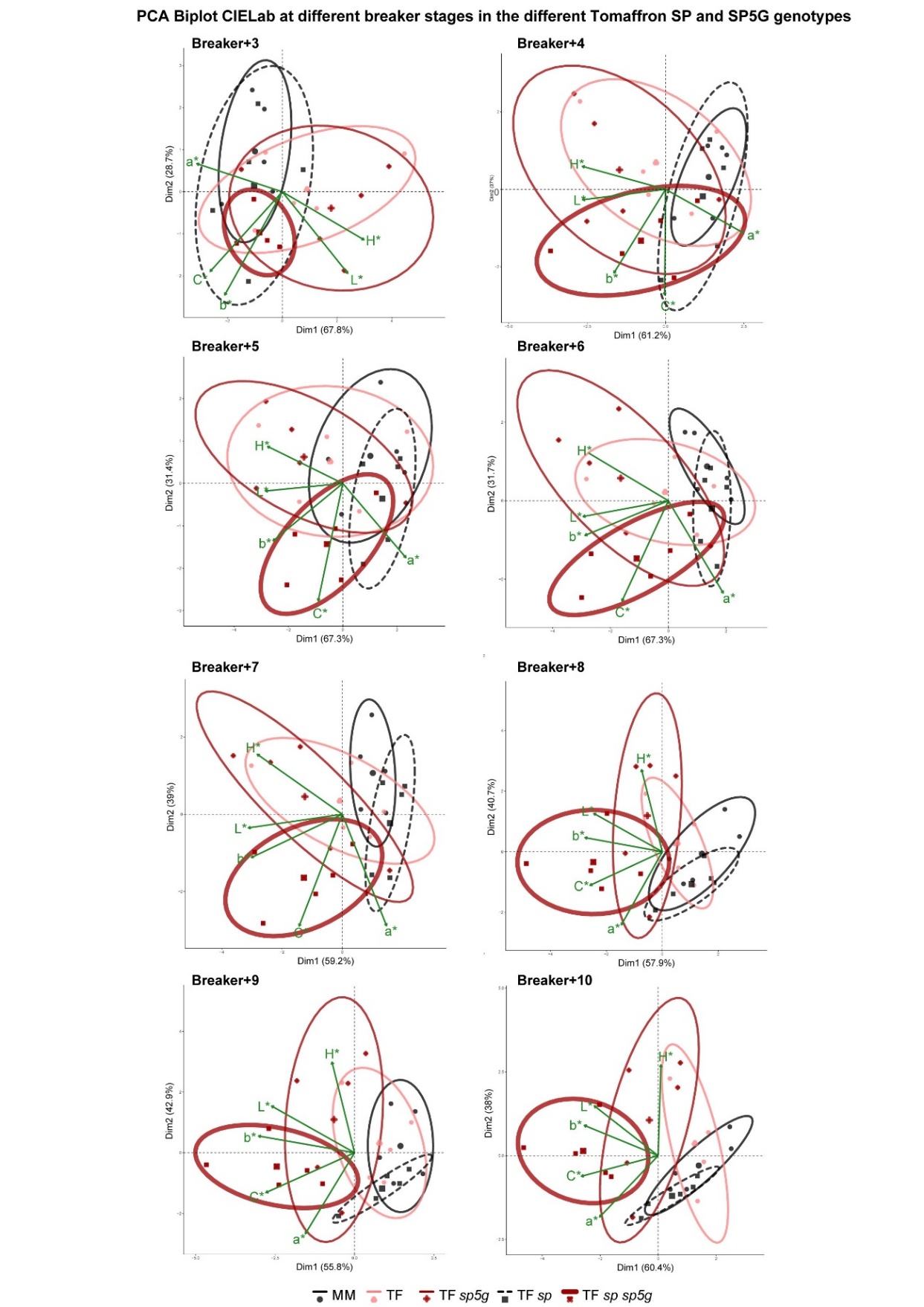


**Supplementary Figure 6**. PCA biplot of the color parameters (C*, L*, a*, b*, H*). Each small symbol represents an individual fruit of each genotype; the big symbol represents the mean of the five fruits. TF: Tomaffron; MM: Moneymaker. *SP*: self-pruning.


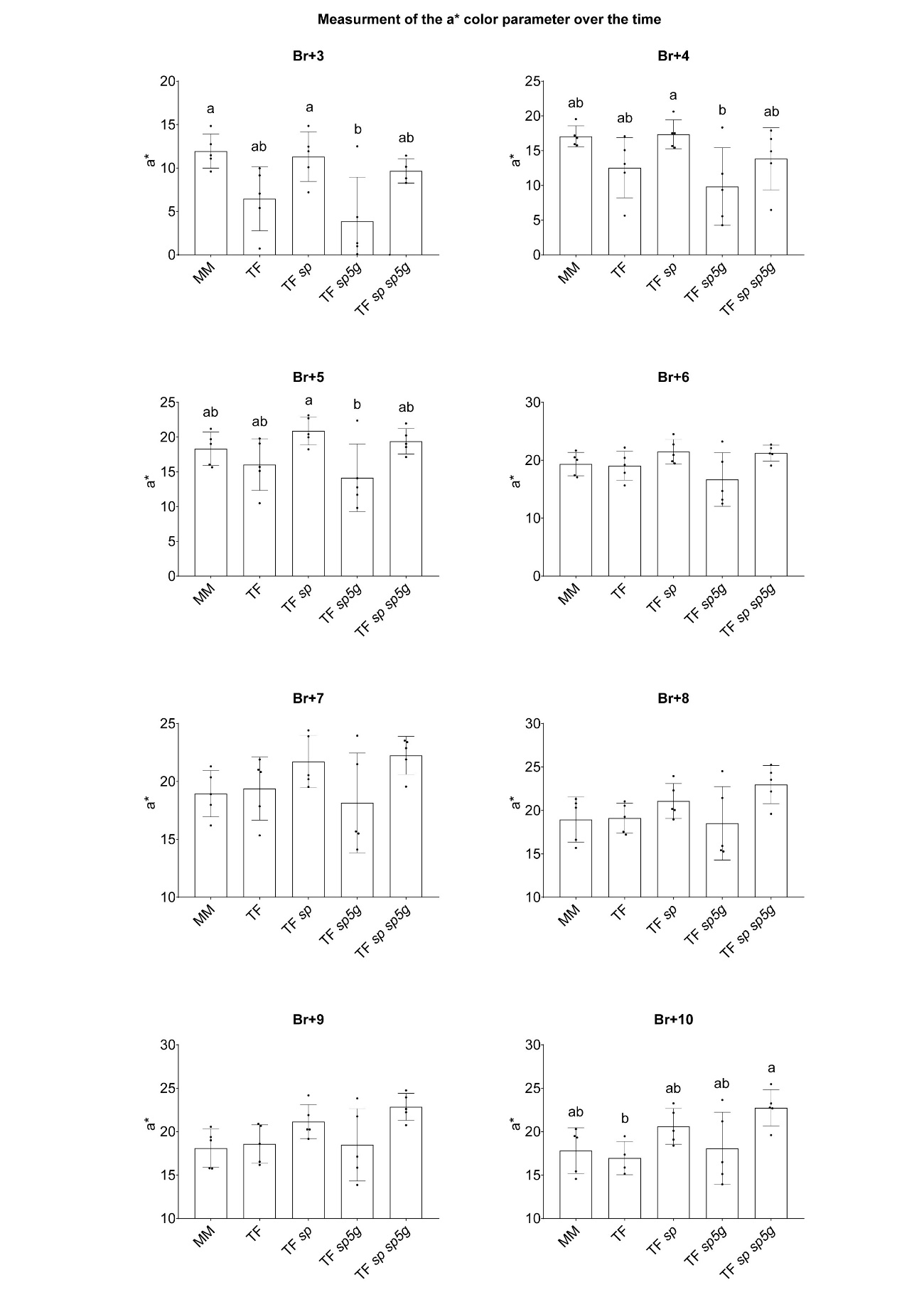


**Supplementary Figure 7**. Changes in the a* color parameter during tomato ripening. The breaker stage is indicated at the top of each graph. Bars represent the mean and the standard deviation, showing individual data points. Group differences were determined using ANOVA followed by Tukey’s post-hoc test (*p* < 0.05). Different letters indicate statistically significant differences. TF: Tomaffron; MM: Moneymaker. *SP*: self-pruning.


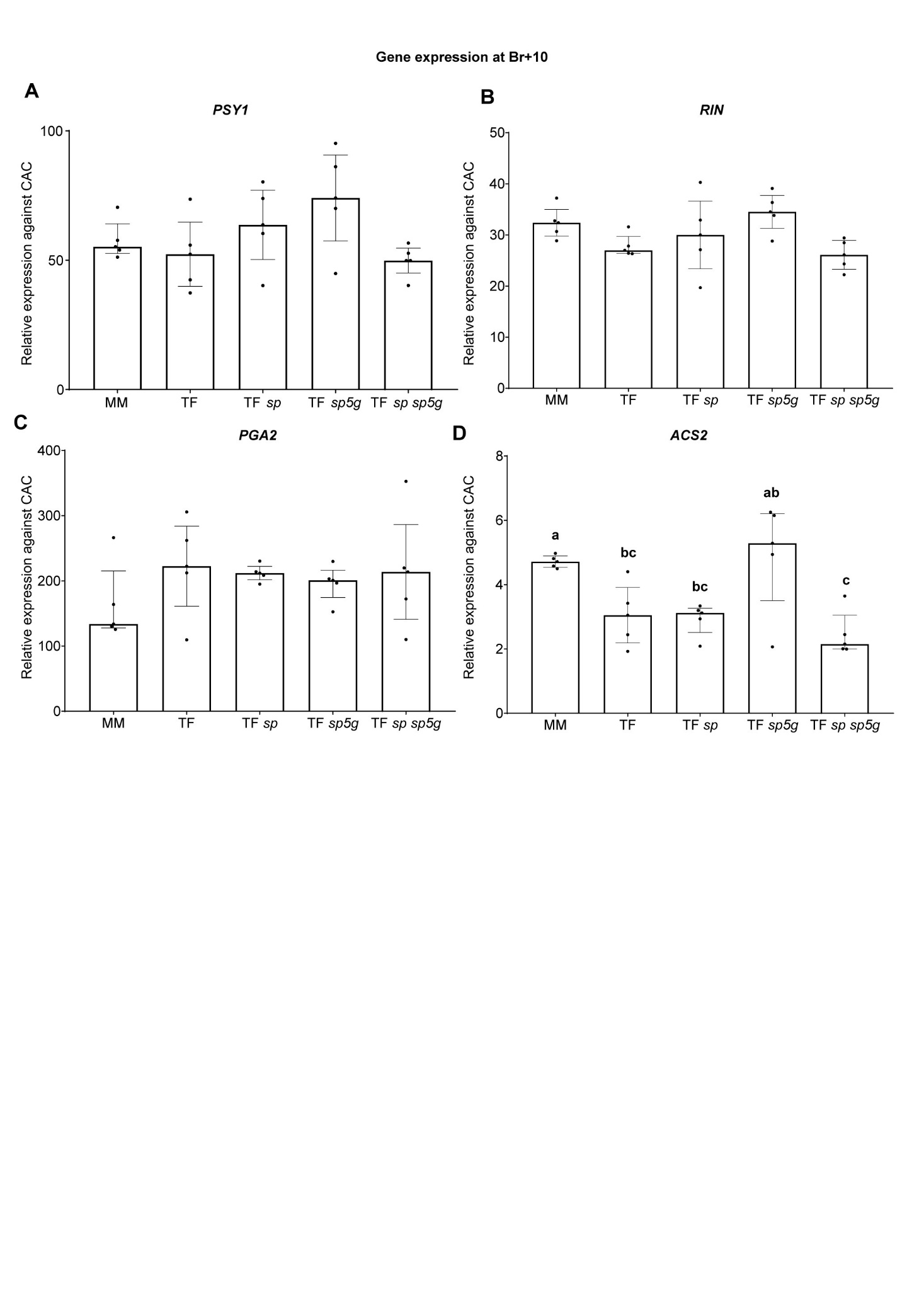


**Supplementary Figure 8**. Gene expression analysis of phytoene synthase 1 (**A**), ripening inhibitor (**B**), polygalacturonase 2A (**C**), and 1-aminocyclopropane-1-carboxylate synthase (**D**) genes measured by RT-PCR using *SlCAC* as the reference gene. Bars represent the median and interquartile range (25th–75th percentile), showing individual data points. Group differences were assessed using a one-way permutation ANOVA followed by pairwise comparisons (*p*-adj < 0.05). Different letters indicate statistically significant differences. TF: Tomaffron; MM: Moneymaker. *SP*: self-pruning.

**
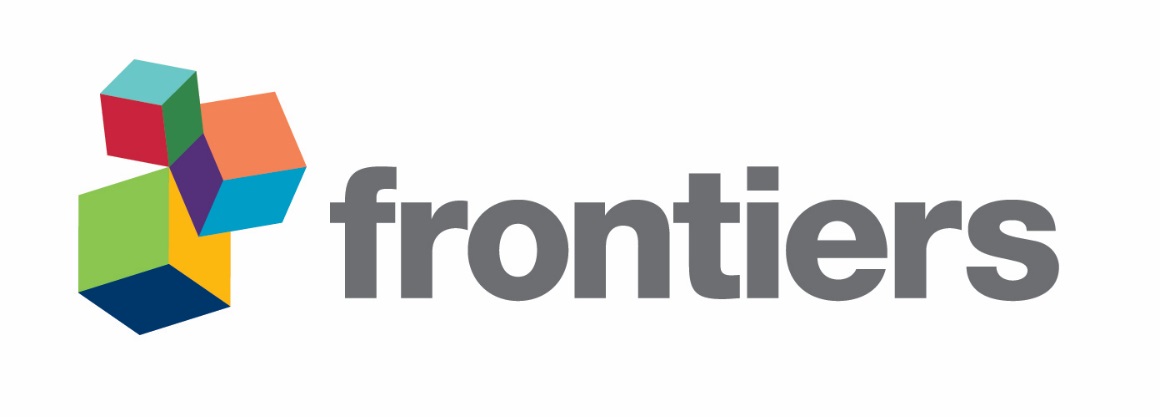
**
